# Conversion therapy strategy: A novel GPC3-targeted multimodal organic phototheranostics platform for mid-late-stage hepatocellular carcinoma

**DOI:** 10.1016/j.mtbio.2024.101442

**Published:** 2025-01-02

**Authors:** Fan Wu, Xin Kuang, Sanlin Deng, Shuo Qi, Jian Xiong, Bibo Zhao, Chuanfu Li, Senyou Tan, Qiang Kang, Hao Xiao, Xiaofeng Tan, Gui-long Wu, Qinglai Yang, Guodong Chen

**Affiliations:** a Department of Hepatopancreatobiliary Surgery, The First Affiliated Hospital, Hengyang Medical School, University of South China, Hengyang, Hunan, 421001, China; b Center for Molecular Imaging Probe Hengyang Medical School, University of South China, Hengyang, Hunan, 421001, China; c Hunan Engineering Research Center for Early Hengyang Medical School, University of South China, Hengyang, Hunan, 421001, China; d Department of General Surgery, Turpan City People's Hospital, Tulufan, 838000, China

**Keywords:** Hepatocellular carcinoma, GPC3, Dual-modality imaging, Phototheranostics, Surgical navigation

## Abstract

Hepatocellular carcinoma (HCC) is typically diagnosed at intermediate to advanced stage, making surgical treatment unfeasible. Conversion therapy aims to reduce tumor stage, improve hepatic resection feasibility, and lower recurrence rates. Since traditional therapies are often accompanied by uncertainty of efficacy, there is an urgent need to explore new treatment strategies. Near-infrared phototheranostics technology provides a new way for HCC diagnosis and treatment by its excellent optical properties. However, complex preparation and poor biocompatibility of phototheranostics probes limit clinical application. In this study, we developed a fluorescence/magnetic resonance dual-modality imaging (FLI/MRI) as well as photothermal/photodynamic therapy (PTT/PDT) GPC3-targeted multifunctional phototheranostics probe, IR820-GPC3-Gd NPs (IGD NPs), to improve the efficiency of conversion therapy for HCC. The IGD NPs were simply prepared with the IR820 as the core. Conjugating the HCC-specific targeting molecule GPC3 peptide and the MRI agent DOTA-Gd through click chemistry. IGD NPs target HCC cells through GPC3, releasing heat and reactive oxygen species (ROS) under noninvasive 808 nm laser irradiation to reduce tumor size and achieve downstaging. High-sensitivity FLI/MRI precisely delineates tumor boundaries, providing real-time surgical navigation and prognosis assessment. This novel probe offers a feasible and effective treatment option for advanced HCC.

## Highlights

• A novel NIR phototheranostics probe IGD-NPs, has been developed based on the excellent optical properties of IR820.
• IGD NPs specifically target HCC cells via GPC3, offering high biocompatibility and safety.
• IGD-NPs enable downstaging of HCC and accurately depict tumor boundaries for real-time surgical navigation.

## Graphical abstract

The water-soluble probe, synthesized using click chemistry to combine IR820, GPC3 peptide, and DOTA-Gd, is employed for PTT/PDT and dual-modality imaging using fluorescence imaging (FLI) and magnetic resonance imaging (MRI) to guide surgical resection, effectively inhibiting mid- to late-stage HCC.

**Figure:**
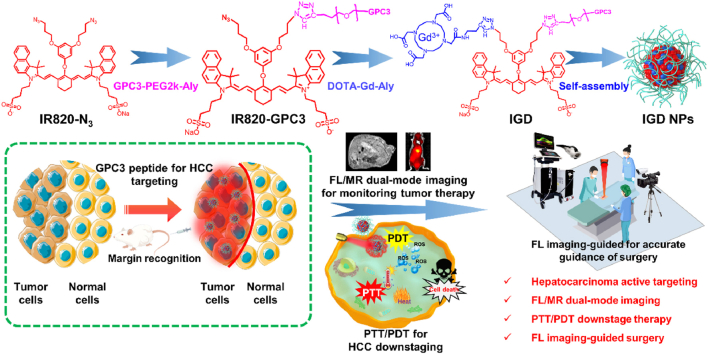

## 1 Introduction

Hepatocellular carcinoma (HCC), as a predominant malignant liver neoplasm, is regularly first diagnosed at a middle or late stage, rendering surgical resection difficult to operate [[1], [2], [3]]. Conversion therapy refers to the conversion of an unresectable tumor into a resectable one (specifically in HCC), followed by surgical removal of the tumor to maximize the survival of patients [4]. The National Comprehensive Cancer Network (NCCN) and Guidelines for the Diagnosis and Treatment of Primary Liver Cancer in China advocate conversion therapy as an initial treatment strategy for unresectable or mid-late-stage tumors to achieve adequate tumor downstaging to undergo surgical resection [[5], [6], [7]]. Combination conversion treatments of molecular targeted therapy, chemotherapy, immunotherapy, and locoregional therapy have been applied in clinical practice and achieved a higher objective response rate and median survival time [8,9]. Despite the significance of the aforementioned synergistic therapy as a key treatment approach for advanced HCC patients, repeated interventions can progressively impair liver function, and conventional treatment protocols often fail to produce the expected outcomes [10]. Therefore, it is necessary to continue to explore new therapeutic approaches to achieve the integration of multiple treatments to ensure a favorable prognosis. Near-infrared (NIR) phototheranostics is a promising modality for diagnosing, monitoring, and treatment of HCC, offering the advantages of efficient phototherapy downstaging and multimodal deep-tissue surgical navigation [[11], [12], [13], [14]], which provides a new conversion therapy way for HCC phototheranostic by its excellent optical properties.

Traditional NIR fluorescence imaging (FLI) provides high sensitivity, rapid response, and non-invasiveness while limited by low resolution and tissue penetration capabilities [14,15]. Integration of other imaging modalities, such as photoacoustic imaging (PAI) and magnetic resonance imaging (MRI), or the developing NIR FLI technology could enhance precise and deep location ability for guideline of tumor surgical resection [[16], [17], [18]]. In addition, the efficacy of single-modality phototherapy is limited by the heat shock effect of photothermal therapy (PTT) and the hypoxic conditions associated with photodynamic therapy (PDT) [[19], [20], [21], [22]]. Combining PTT and PDT represents an efficient approach because PTT can enhance vascular permeability and sensitize tumor tissues to the oxidative stress induced by PDT [23,24]. Therefore, the development of multifunctional NIR phototheranostics with simultaneous multimodal imaging and synergistic phototherapy is warranted. For example, Wang et al. developed an HCC-specific contrast agent, IPP-c, to facilitate minimally invasive PTT/PDT treatment guided by FLI/PAI to prevent harm to surrounding tissues [25]. Liu et al. prepared multifunctional lipid micelles for enhanced MRI/PAI imaging and bimodal PDT/PTT therapy to enhance the efficacy of HCC treatment [26]. Chen et al. introduced a NIR-II aggregation-induced emission-based luminogen (AIEgen) photosensitizer for assisting in tumor surgery and achieving tumor reduction through phototherapy [27]. Despite the progress made in the study of phototheranostics probes for HCC, the difficulty in combining efficient optical performance and excellent biocompatibility has limited their therapeutic effectiveness [28]. Therefore, there is an urgent need to develop an efficient and biocompatible multimodal phototheranostics probe to enhance the efficiency of conversion therapy with urgency.

Indocyanine green (ICG), as one of the NIR imaging contrast agents approved by the U.S. Food and Drug Administration (FDA) for clinical application, has been utilized in liver reserve function assessment and surgical guidance due to its favorable biocompatibility [[29], [30], [31]]. Nonetheless, ICG is hindered by inadequate photostability, low photothermal conversion efficiency (PCE), and suboptimal tumor targeting, thereby confining its utility in deep and high-precision HCC surgical navigation [32]. New ICG derivatives, like IR820, are seen as promising alternatives to conventional ICG due to their enhanced optical properties and structural flexibility, enabling diverse clinical applications [33,34]. Glypican-3 (GPC3) is a sensitive biomarker of HCC with elevated expression compared with that in normal liver tissues [35,36], which could be a powerful tool for modification of ICG derivative phototheranostics to enhance the precision of imaging and treatment.

**Scheme 1:**
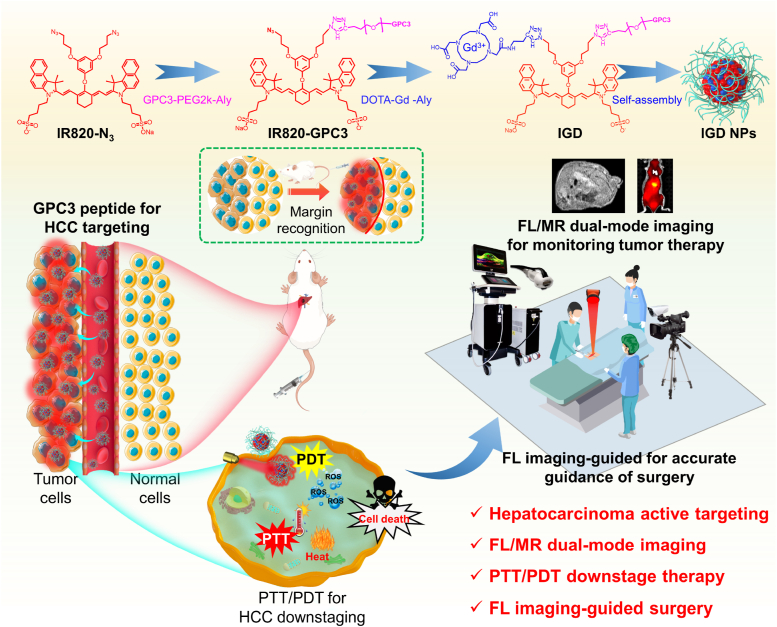
Multifunctional phototheranostics probe IGD NPs for the phototherapy and surgical resection of HCC.

Herein, a GPC3-targeted multifunctional phototheranostics, IR820-GPC3-Gd NPs (IGD NPs), was developed for conversion therapy of HCC (Scheme 1). IGD was rationally designed from IR820 derivative with modification of azide group for coupling with GPC3 peptide and DOTA-Gd by click chemistry. Subsequently, the amphiphilic IGD molecules were self-assembled into IGD NPs by nonparticipation method for better tumor accumulation by GPC3-targeted. The IGD NPs exhibited a good active targeting ability to HCC to benefit the endocytosis of nanoparticles. Under noninvasive 808 nm laser irradiation, the IGD NPs exhibit an excellent PTT/PDT for downstaging unresectable HCC. Subsequently, the combinational NIR FLI and MRI ability of IGD NPs allows for precise localization of residual tumor boundaries and real-time surgical navigation, ultimately leading to significantly low postoperative recurrence rates. This work provides insight into the design of potential phototheranostics to improve the conversion therapy efficiency of HCC and offers an effective strategy for tumor downstaging therapy and surgical navigation.

## 2 Materials and methods

### 2.1 Materials and reagents

GPC3 peptide and DOTA-Gd were purchased from Chinapeptides Co., Ltd, the Cell Counting Kit (CCK8), AM/PI staining kit, 2′,7′-dichlorofluorescein diacetate (DCFH-DA) was purchased from Abbkine Scientific Co., Ltd, M Matrigel, and D-Luciferin potassium salt were purchased from Shanghai Zhongqiao Xinzhou Biological Technology Co., Ltd. Chemicals for Organic Synthesis Ordered from Shanghai Titan Technology Co., Ltd. HepG2-luc cell (Human Hepatocellular Carcinoma Cell Line-Luciferase Labeled) was obtained from ATCC (American type culture collection). DMEM (Dulbecco's Modified Eagle Medium, Dulbecco's), trypsin, and fetal bovine serum were purchased from Dalian Meilun Biotechnology Co. All investigations involving animals in this work received approval from the Animal Ethics Committee of the Department of Laboratory Animal Science at the University of South China (ethics number: 2023027).

### 2.2 *In vitro* photothermal and ROS assay

Ultrapure aqueous solutions of IGD NPs at various concentrations (0, 25, 50, 75, 100 μM) were irradiated with an 808 nm laser (1.0 W/cm^2^) for 10 min, with thermal images captured every 2 min. Laser power (0.25, 0.5, 0.75, 1.0 W/cm^2^) was varied to determine the optimal probe concentration and power settings for future experiments and compared with IR820. Solutions of IGD NPs and IR820 (75 μM) with 2′,7′-dichlorofluorescein diacetate (DCFH-DA) were irradiated under the same laser conditions at five-time points (0, 2.5, 5.0, 7.5, 10 min), and fluorescence at 525 nm was recorded under 488 nm excitation.

### 2.3 *In vitro* fluorescence and MR signals of IGD NPs

Prepare 200 μL of ultrapure water solution of molecular probe IGD NPs with different concentrations (0, 25, 50, 75, 100 μM) and add it into the EP tube. The EP tube was placed into the elevator of the *in vivo* imaging system for small animals (IVIS) to obtain images and quantitative fluorescence values. The EP tube was placed into the center of the magnetic field of the 3.0 T MRI. T1 relaxivity measurements and T1-weighted MRI at 25 °C. The relaxivity values of *r*_*1*_ were calculated by fitting the 1/T1 relaxation time (s^−1^) versus the IGD NPs concentration (mM) curves.

### 2.4 Cell uptake experiments

The experiment was divided into IR820 and IGD NPs groups. Hep-G2 cells (2 × 10^5^ cells/dish) grown in the logarithmic phase were selected and inoculated into colocalization for culture. 12 h later, the supernatant was washed off, and the probe (75 μM) was added and co-incubated with the cells for 0, 3, and 6 h. Subsequently, the probe uptake was observed under confocal laser scanning microscopy (CLSM).

### 2.5 Live/dead cells staining

To assess nanoparticle toxicity, a live/dead cell staining assay was conducted. Hep-G2 cells (3 × 10⁵ cells/dish) were cultured in Dulbecco's modified eagle medium (DMEM) with 10 % FBS and 1 % PS at 37 °C with 5 % CO₂. After overnight incubation, the medium was replaced with fresh solutions containing various treatments (PBS, PBS + NIR, IGD NPs, IGD NPs + NIR) at 75 μM concentration. After 4 h of incubation, cells were rinsed with PBS, stained with a Living/Dead cell double staining kit (Calcein AM/PI), and analyzed by CLSM.

### 2.6 CCK8 assay

Nanoparticle cytotoxicity was assessed via the Cell Counting Kit (CCK8) assay. Hep-G2 cells (5000 cells/well) were cultured for 24 h and then treated with IGD NPs at different concentrations for 4 h. Following treatment, cells were irradiated with a laser for 10 min. After a further 24 h in the dark, cell viability was measured using the CCK8 assay.

### 2.7 Intracellular ROS detection

To assess ROS generation, DCFH-DA was used. Hep-G2 cells (2 × 10⁵ cells/mL) were cultured overnight and then treated with 75 μM nanoparticles for 4 h. After washing with PBS, the cells were treated with DCFH-DA (10 μM) for 30 min. CLSM analysis was performed with excitation at 488 nm and emission was collected between 490 and 550 nm.

### 2.8 Establishment of Hep-G2 tumor models

Hep-G2 cells (1 × 10⁷ cells/mL) were injected subcutaneously into the left thigh of mice to create a tumor model. Tumor growth was monitored regularly, and further experiments were conducted once the tumor reached approximately 80 mm³. Similarly, Hep-G2 cells (2 × 10^7^ cells/mL) were injected subcutaneously under the peritoneum of the lower segment of the left outer lobe of the liver in mice to establish an in situ transplantation tumor model, and the tumor was determined by intraperitoneal injection of the substrate D-Luciferin potassium salt, and bioluminescence assay was carried out under the IVIS to determine whether or not the tumor had become a tumor. Follow-up experiments were performed when the tumor grew to about 50 mm^3^.

### 2.9 FLI/MRI of mice

Eight mice with in situ tumors were randomly assigned to two groups and administered 50 μL of 200 μM IGD NPs or ICG via the tail vein, respectively. Imaging was conducted via the IVIS equipment at 0.5, 4, 8, 12, 24, and 48 h. After 24 h, the mice were euthanized, and their organs and tumors were collected for FLI. The tumor's region of interest (ROI) was outlined by the software to quantify and identify the peak probe accumulation time. Four mice with subcutaneous tumors were injected via the tail vein with 100 μL of 200 μM IGD NPs and an equal volume of PBS. They were then anesthetized with the tumor positioned in the magnetic field. MRI and T1-weighted signal intensities were measured at 0, 24, and 48 h. This procedure was repeated for imaging of the subcutaneous tumors.

### 2.10 Phototherapy of subcutaneous tumor mice

Mice were categorized into three groups: PBS, ICG, and IGD NPs, with four mice in each group. After injecting the materials via the tail vein, tumors were irradiated with an 808 nm laser (1.0 W/cm^2^, 10 min) at the optimal time. Body weight and tumor volume were recorded every three days to assess treatment effects and tumor recurrence, with volumes calculated using a specific formula.

$$Tumor\;volume\left(V\right)=\frac{W^{2}\times L}{2},Relative\;tumor\;volume=\frac{V}{V0}$$

### 2.11 Non-invasive phototherapy and surgical navigation in mice with in situ tumors

Mice were categorized into two groups: PBS and IGD NPs, with four mice in each group. Following tail vein injections, the mice were anesthetized and positioned supine with the abdomen exposed. Tumors were then irradiated using an 808 nm laser (1.0 W/cm^2^, 10 min). Once the mice were stabilized, tumors were surgically removed at a subsequent time. After the tumor location was determined by preoperative MRI/FLI, a longitudinal skin incision of approximately 1 cm in length was made with a scalpel to expose the liver tumor in the mice. The liver tumor border was determined under FL guidance, and the site was radically resected with a surgical electrocoagulation knife until there was no remnant of any tumor fluorescence signal, indicating a negative hepatic incision margin. The incision site was sutured and sterilized. Mouse vital signs were monitored throughout the operation. Tumor bioluminescence signals were recorded at each stage, and the mice were weighed. After treatment, the naked mice were euthanized, and the tumor tissue was excised and photographed for documentation purposes.

### 2.12 Pathologic analysis

Tissues removed surgically were subjected to staining with hematoxylin and eosin (H&E), Ki-67, and TUNEL. On the 15th day post-treatment, the mice were euthanized, and blood samples, including whole blood and serum, were collected for routine blood tests and evaluation of liver and kidney functions. Additionally, vital organs and tumor tissues were excised, stained with H&E, and examined under a light microscope to assess their morphology and structure.

### 2.13 Statistical analysis

Statistical analysis was conducted using two-tailed paired sample Student's t-tests in SPSS 26.0. Results are expressed as mean ± standard deviation (SD) from experiments conducted in triplicate or more. Significance was determined with a threshold of *p* < 0.05, with distinctions denoted as ∗ *p* < 0.05, ∗∗*p* < 0.01, and ∗∗∗*p* < 0.001.

## 3 Results and discussion

### 3.1 Synthesis and characterization

The synthesis procedures for the phototheranostics IR820-GPC3-Gd NPs (IGD NPs) have been presented detailed in Scheme S1. The structural analysis of IGD NPs and their precursors was performed using nuclear magnetic resonance (NMR), high-resolution mass spectrometry (HRMS), and Size exclusion chromatography (SEC) (Figs. S1–S10).

**Fig. 1:**
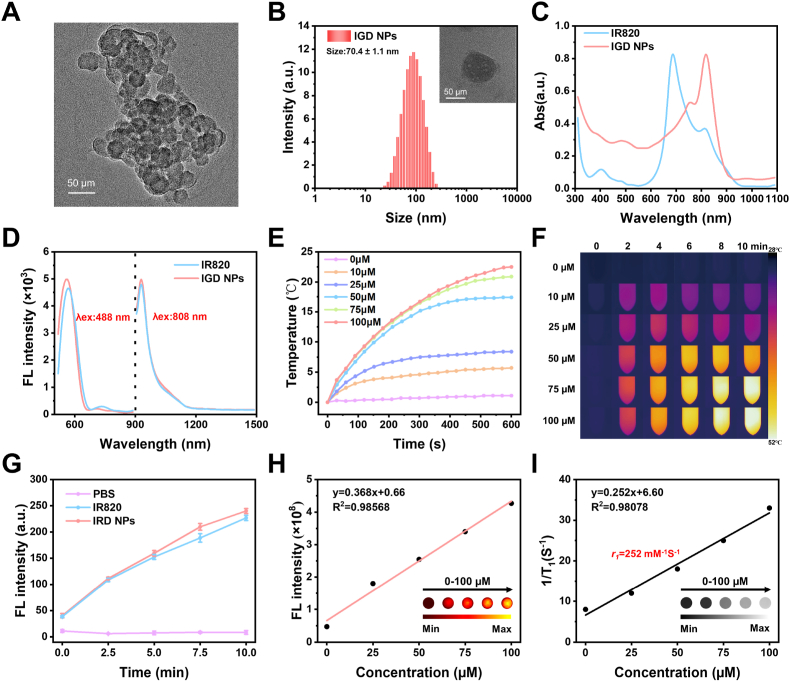
Physicochemical characterization of IGD NPs. (A) TEM image of IGD NPs. (B) Hydrated particle size map of IGD NPs, scale bar: 50 nm. (C) Absorption spectra of IR820 and IGD NPs in the aqueous phase. (D) Fluorescence spectra of IR820 and IGD NPs with excitation of 488 nm and 808 nm, respectively. (E, F) Photothermal warming curves and thermograms of IGD NPs at different concentrations. (G) After laser irradiation at different times, the DCF fluorescence values in aqueous solution of PBS, IR820, and IGD NPs (488 nm). (H, I) Linear fitting curves of fluorescent and magnetic resonance signals of IGD NPs aqueous solution at different probe concentrations, with corresponding FLI and MRI in the insets.

The results from SEC analysis demonstrate that the retention time was reduced upon coupling with various moieties (Fig. S11). The observed *M*_*w*_ differences, specifically in GPC3-PEG2000 (2900), IR820-GPC3 (4100), and IR820-GPC3-Gd (4700), suggest that different moieties were stably connected (Table S1). Transmission electron microscopy (TEM) and dynamic light scattering (DLS) were employed to examine the morphology and size distribution of the IGD NPs. The results indicated that IGD NPs displayed a spherical-like configuration with an average diameter of 70.4 ± 1.1 nm and a Zeta potential of −16.1 ± 0.49 mV (Fig. 1A and B, and S12). Compared to the commercial IR820, IGD NPs exhibited a red-shifted absorption with a maximum peak at 810 nm (Fig. 1C). Furthermore, IGD NPs inherited the dual emission characteristics of IR820 with peaks at 930 nm (λex:808 nm) and 560 nm (λex:488 nm) (Fig. 1D). Further, heat and ROS generation of IGD NPs were evaluated. The IGD NPs exhibited higher photothermal conversion ability than that of IR820 with a temperature rise of 21 °C under the 10 min laser irradiation (Fig. S13A). The IGD NPs presented a concentration-dependent photothermal performance with fast heating to 45 °C at a low concentration of 75 μM, which is enough to effectively kill tumor cells (Fig. 1E and F) [37,38]. Similarly, the temperature of IGD NPs would rise with increasing laser power (Fig. S13B). The photodynamic efficacy of IGD NPs was assessed using the ROS probe 2′,7′-dichlorofluorescein diacetate (DCFH-DA), which is quickly oxidized by reactive oxygen species (ROS) to produce fluorescent dichlorofluorescein (DCF) [39]. Upon exposure to an 808 nm laser, IGD NPs exhibited peak DCF fluorescence at 525 nm, signifying the release of ROS from the nanoparticles, with the fluorescence intensity increasing for the irradiation (Fig. 1G and S14). The above results indicate that IGD NPs have excellent photothermal and photodynamic therapeutic potential.

The FLI and MRI capabilities of IGD NPs were subsequently measured. The FL imaging signal was gradually linearly enhanced with the increase of IGD NPs concentration (R^2^ = 0.98568) (Fig. 1H). Moreover, IGD NPs were unaffected by modifiers and maintained high fluorescence stability. Meanwhile, the T1-weighted signal also gradually increased with increasing concentration of the IGD NPs due to the chelation of DOTA-Gd (R^2^ = 0.98078) (Fig. 1I). The IGD NPs showed good magnetic resonance paramagnetic properties with an *r*_*1*_ relaxation rate of 252 mM^−1^S^−1^. Thus, the sensitive fluorescence and magnetic resonance imaging signals of IGD NPs demonstrate significant potential for imaging and localization in HCC.

### 3.2 Influence of IGD NPs on Hep-G2 cells

**Fig. 2:**
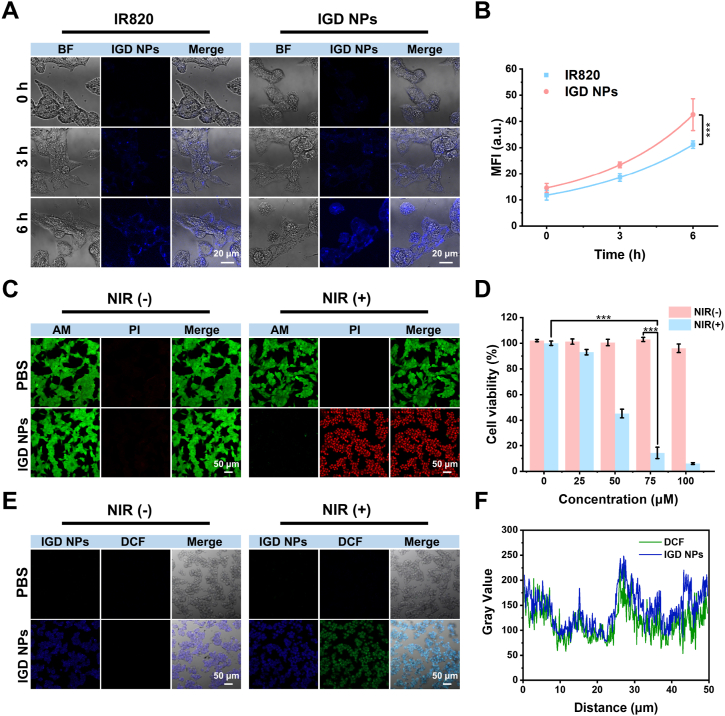
Targeting ability, toxicity, and treatment of IGD NPs in Hep-G2 cells. (A, B) Cellular uptake plots and fluorescence values of IR820 and IGD in Hep-G2 cells at different time points; scale bar: 50 μm, Error bars: mean ± SD (n = 3). (C) Cell viability of Hep-G2 cells observed by live/dead staining tests assay (green for live cells, red for dead cells). (D) Effect of different concentrations of IGD probes on Hep-G2 cell activity under laser irradiation and no irradiation. (E) Detection and quantification of ROS levels in Hep-G2 cells across various treatment groups. Scale bar: 50 μm, Error bars: mean ± SD (n = 3). (F) Intensity distribution of the linear region of interest between DCF and IGD NPs, ∗∗∗*p* < 0.001.

The influence of IGD NPs on the HCC cells was evaluated using the Hep-G2 cell line. The uptake of IGD NPs by Hep-G2 cells was observed using a confocal laser scanning microscope (CLSM). The results showed that the fluorescence intensity of cells co-incubated with the IGD NPs enhanced gradually with increasing time than that of IR820-treated cells at the same conditions (Fig. 2A and B), suggesting that the modification of GPC3 effectively enhanced the uptake of IGD NPs in HCC cells [40,41].

To assess the tumor cell-killing efficacy of IGD NPs, live/dead staining was conducted using Calcein Acetoxymethyl Ester (Calcein AM, which stains live cells green) and Propidium Iodide (PI, which stains dead cells red). Significant green fluorescence with almost no red fluorescence was observed in the PBS-treated and IGD NPs-treated groups, which demonstrated that most cells survive without laser irradiation. However, after irradiation with an 808 nm laser, IGD NPs induced a large number of Hep-G2 cell deaths owing to the release of an amount of heat and ROS (Fig. 2C). The cytotoxic effects of various concentrations of IGD NPs on Hep-G2 cells were assessed using the Cell Counting Kit 8 (CCK8) assay. Findings revealed that cell viability remained above 90 % at concentrations up to 100 μM in the absence of laser irradiation, suggesting that the dark toxicity of IGD NPs is minimal (Fig. 2D). However, after 808 nm laser irradiation, IGD NPs exhibited significant tumor cell-killing ability. When the IGD NPs concentration was 75 μM, the cell viability decreased significantly to 14.4 %, showing the potent tumor cell-killing ability of IGD NPs with laser irradiation.

DCFH-DA was employed to monitor the production of intracellular ROS following various treatments. Fig. 2E illustrates that minimal green fluorescence was observed in the PBS and IGD NPs groups without laser exposure. Conversely, upon irradiation with an 808 nm laser, a marked increase in green fluorescence was detected in cells treated with IGD NPs, suggesting that these nanoparticles effectively generated substantial ROS levels within the cells, leading to tumor cell death [42]. The fluorescence colocalization results also confirmed that IGD NPs produce large amounts of ROS within the cell (Fig. 2F).

### 3.3 FLI and MRI of IGD NPs *in vivo*

**Fig. 3:**
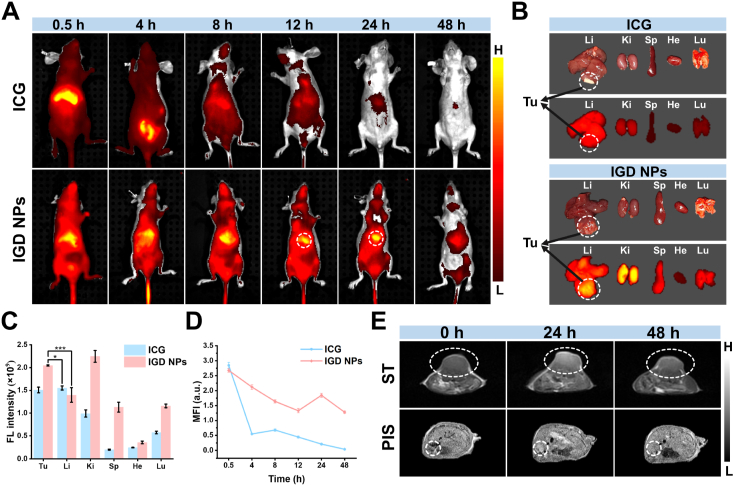
FLI and MRI of IGD NPs in Hep-G2 tumor-bearing mice. (A, C) The FLI and signal values *in vivo* of in situ tumor-bearing mice were measured at various time points after tail vein injection of free ICG and the IGD NPs probe. Error bars: mean ± SD (n = 4). (B, D) FLI and signal values of tumors and major isolated organs 24 h after intravenous injection of free ICG and IGD NPs probes. Error bars: mean ± SD (n = 4). Error bars: mean ± SD (n = 4). (E) T1-weighted MRI of mice after tail vein injection of IGD NPs probe at different time points. ST: subcutaneous tumors, PIS: in situ tumors. ∗*p* < 0.05, ∗∗∗*p* < 0.001.

Both subcutaneous and in situ Hep-G2 tumor models were successfully established for subsequent animal imaging experiments. First, the FLI ability of free ICG and IGD NPs was investigated *in vivo* in mice. After tail vein injection, IGD NPs were rapidly distributed throughout the body through blood vessels and highly aggregated in liver tissue, with low contrast between tumor and normal liver tissue (Fig. 3A and C). With time, the fluorescence signal of normal tissue decreases and the tumor region remains high until 24 h with the best contrast. Unlike IGD NPs, the metabolism and degradation patterns of ICG showed significant differences. Following ICG injection, fluorescence signals quickly concentrated in the liver tissue, then diminished in the abdominal area, disappearing entirely after 4 h without any accumulation in the tumor site. The fluorescence distribution in subcutaneous tumor-bearing mice mirrored the findings observed in in situ tumor-bearing mice (Fig. S15). 24 h after injection, FLI was performed on the major organs and tumors of mice treated with ICG and IGD NPs. As shown in Fig. 3B and D, fluorescence signals were predominantly localized in the liver, kidneys, and tumors. While the ICG group showed no notable difference in fluorescence between the liver and tumor tissue, the IGD NPs group exhibited significantly higher fluorescence intensity in the tumor compared to the normal liver tissue, with a distinct boundary visible. This was a result of the successful introduction of active targeting GPC3 peptide into IGD NPs to cause the long-term enrichment and retention of IGD NPs in HCC tissues with stronger fluorescence signals [43].

Given the substantial MRI potential of IGD NPs, we conducted a more thorough assessment in the Hep-G2 tumor-bearing mice. In both subcutaneous and autochthonous tumors, a highly T1-weighted MRI signal peak was observed 24 h post-injection and persisted for more than 48 h (Fig. 3E and S16). These results suggest that IGD NPs have exceptional MRI capabilities. The dual-mode FLI/MRI features with extended *in vivo* cycle time enabled multimodal imaging analysis for HCC, thereby providing a longer imaging window for precise surgical localization and navigational resection.

### 3.4 Phototherapy downstage in subcutaneous tumor-bearing mice

**Fig. 4:**
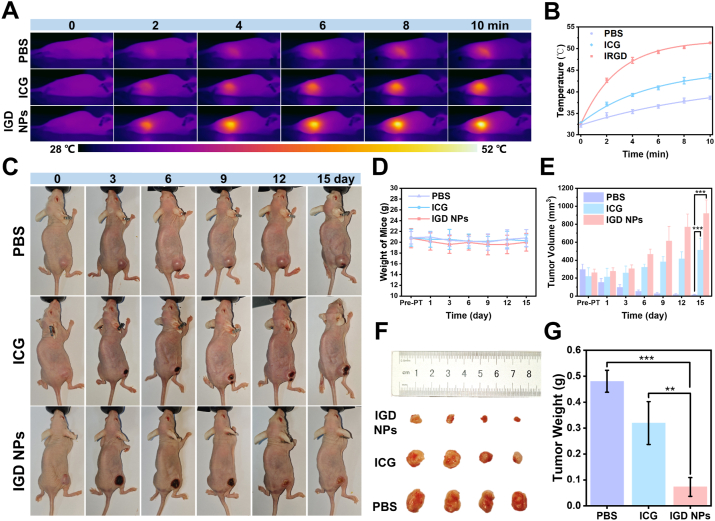
Evaluation of IGD NPs phototherapy in subcutaneous tumors. (A, B) Thermal imaging and temperature variation curves at various time intervals during 808 nm laser irradiation were recorded following the tail vein injection of PBS, free ICG, and the IGD NPs probe. (C) Photographs of tissue changes of subcutaneous tumors in different groups within 15 days after phototherapy. (D, E) Tumor volume and weight change curves in different groups after 15 days of phototherapy. (F, G) Photographs of different groups of tumors and weight differences after 15 days. Error bars: mean ± SD (n = 4), ∗∗p < 0.01, ∗∗∗p < 0.001.

IGD NPs demonstrated exceptional photothermal and photodynamic properties against tumor cells, which further facilitates the investigation of its anti-tumor effects *in vivo*. Hep-G2 subcutaneous tumor-bearing nude mice were categorized into three groups: PBS, ICG, and IGD NPs. These groups were subjected to 808 nm laser irradiation for 10 min to monitor temperature variations. The findings demonstrated a rapid and substantial temperature rise in the tumors of the IGD NPs group, surpassing 51 °C within the 10-min period, which effectively ablated the tumor while minimizing harm to surrounding healthy tissues [44]. In contrast, the ICG group showed a slower temperature increase with the temperature rising to 44.0 °C after 10 min. The tumor region in the PBS group exhibited a significantly slower temperature increase, reaching an average of only 38.8 °C after 10 min, which is well below the threshold necessary for effective tumor ablation (Fig. 4A and B).

The assessment of the therapeutic effect on subcutaneous tumors after phototherapy is a critical step in measuring the efficacy of IGD NPs *in vivo*. The tumor volume and weight of each tumor were examined every 3 days for a total of 15 days. The results in Fig. 4C–E displayed that the IGD NPs group exhibited a substantial therapeutic effect, as evidenced by a substantial reduction in tumor volume and weight, meaning a significant inhibition of lesions with laser irradiation. In contrast, the ICG group exhibited a minor ablation effect, which was accompanied by a slight inhibition of tumor growth. After 15 days, subcutaneous tumors were taken for morphology observation, which further verified the tumor inhibition effect of the IGD NPs group (Fig. 4F and G). Effective synergy of PTT and PDT is the key to shrinking tumor volume and reducing tumor activity [45]. Utilizing the optical therapeutic advantages of IGD NPs promotes tumor downstaging and creates favorable conditions for subsequent surgical resection.

### 3.5 Conversion therapy of IGD NPs in mice with in situ tumors

**Fig. 5:**
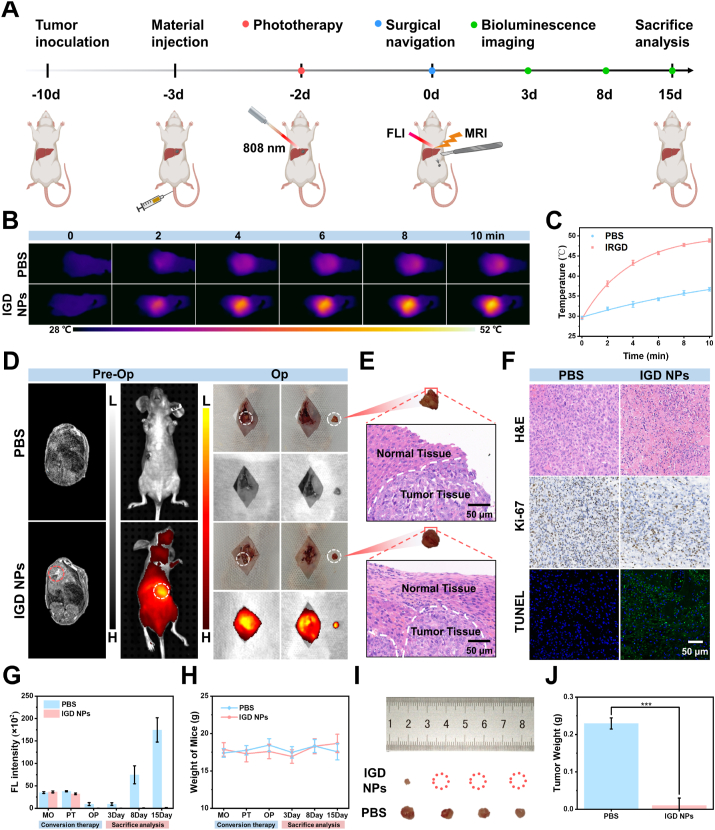
Noninvasive phototherapy and navigated surgical resection of IGD NPs in mice with tumors in situ tumors. (A) Timeline of conversion therapy in mice with IGD NPs in situ tumors. (B, C) Thermal imaging and temperature variation curves at various time intervals during 808 nm laser irradiation were recorded following the tail vein injection of PBS and the IGD NPs probe. (D) Preoperative FLI/MRI localization and FLI-guided surgical resection in mice from different treatment groups. (E) H&E staining results of resected tissue edges in different treatment groups. Scale bar: 50 μm. (F) H&E staining, Ki-67, and TUNEL fluorescence staining results of tumor tissues in different treatment groups. Scale bar: 50 μm. (G) Bioluminescence imaging before and after treatment to continuously monitor the fluorescence signal values of in situ liver tumors in different groups. (H) Weight change curves of different groups before and after treatment. (I, J) Photographs of different groups of tumors and weight differences after 15 days. Error bars: mean ± SD (n = 4), ∗∗∗*p* < 0.001.

The systematic conversion therapy for in situ tumor mice was further performed based on IGD NPs. Hep-G2 tumor-bearing mice were divided into PBS and IGD NPs groups (n = 4) with laser irradiation for HCC downstaging, guided surgical resection, and surgical evaluation (Fig. 5A). 24 h after the tail vein injection of IGD NPs, tumors were identified using FLI and subsequently exposed to 808 nm laser irradiation for phototherapy downstaging. The findings demonstrated that the tumor temperature in the IGD NPs group quickly rose to 48.4 °C within 10 min, successfully achieving *in vivo* photothermal tumor ablation. In contrast, the tumor temperature in the PBS group rose more slowly, with an average temperature of 36.2 °C after 10 min (Fig. 5B and C).

After phototherapy, the mice were monitored closely until they reached a stable condition for surgical resection. The surgical procedures are shown in Fig. 5D and S17. Briefly, 24 h before surgery, IGD NPs were injected into the mice through the tail vein, and the boundaries of the residual tumor were precisely defined using FLI and MRI. Intraoperatively, the tumor tissue was successfully and completely resected under FLI guidance, while the residual tumor tissue under the guideline of FLI was cleared. Subsequently, the tumor margins of resected tissues were examined using H&E staining. The findings revealed that the IGD NPs group exhibited clean margins with a distinct separation between tumor and normal tissues, in contrast to the PBS group, which showed unclear tumor margins (Fig. 5E). Tumor tissues were further analyzed through H&E staining, Ki-67, and TUNEL staining. The findings demonstrated that tumor tissues in the IGD NPs group exhibited necrosis, with the lowest levels of Ki-67 positivity and the highest TUNEL signals, indicating that IGD NPs effectively triggered tumor cell death following 808 nm laser irradiation (Fig. 5F).

Bioluminescence was implemented to monitor tumor growth throughout the treatment (Fig. 5G and S18). The IGD NPs group demonstrated a significantly superior treatment efficacy in comparison to the PBS group, as the tumors in the IGD NPs group contracted after two days of laser treatment, whereas the tumors in the PBS group persisted in their expansion. Tumor recurrence in mice was assessed on days 3, 8, and 15 following surgery. The IGD NPs group underwent clean resection, and no tumor signals were observed. However, the PBS group exhibited significant fluorescent signals after surgery on day 8, indicating that all rodents had tumor recurrence due to incomplete resection. The body weight of the mice did not change significantly throughout the treatment (Fig. 5H). At 15 days, the mice were executed, and the tumor tissues, blood, and all major organs (heart, liver, spleen, lungs, and kidneys) were removed for analysis. Blood was used for routine hematological and biochemical assays, and all organs were stained with H&E staining. The results showed that only one tiny tumor was found in the IGD NPs group, while larger tumor recurrence was observed in all PBS groups (Fig. 5I and J). Routine hematological and biochemical assays were normal between the PBS and IGD NPs treatment groups (Fig. S19). H&E staining results indicated that both groups showed no substantial damage or notable abnormalities in their vital organs (Fig. S20), suggesting that IGD NPs have good biosafety. The results suggest that IGD NPs have a safe and effective phototherapeutic intervention for in situ liver tumors, as well as complete resection of tumor tissues under FLI guidance to reduce the postoperative recurrence rate.

## 4 Conclusion

In summary, a new HCC-specific targeted phototheranostics probe IGD NPs were effectively constructed. IGD NPs demonstrated a strong targeted uptake ability in Hep-G2 cells, ensuring effective accumulation in tumor cells while minimizing nonspecific distribution to normal tissues, while also demonstrating excellent biosafety. Tests revealed that the probe exhibited exceptional physicochemical features, FLI, and MRI monitoring capabilities, with imaging signals that persisted for over 48 h. IGD NPs generated heat and ROS that had substantial lethal effects on tumor cells under laser irradiation. The synergistic effect of PTT and PDT effectively ablated the tumors and accomplished downstaging in a mouse model of HCC. Following laser treatment, the postoperative recurrence rate was substantially reduced as a result of the precise localization of residual tumor boundaries and real-time surgical navigation of resection guided by FLI through the use of FLI and MRI. In conclusion, the IGD NPs probe's multifunctional properties of FLI/MRI and PTT/PDT render it a viable solution for conversion therapy and surgical navigation of HCC.

## CRediT authorship contribution statement

**Fan Wu:** Writing – review & editing, Writing – original draft, Visualization, Validation, Supervision, Investigation, Formal analysis, Data curation, Conceptualization. **Xin Kuang:** Software, Resources, Investigation, Funding acquisition, Formal analysis. **Sanlin Deng:** Visualization, Software, Investigation, Funding acquisition, Formal analysis, Data curation. **Shuo Qi:** Supervision, Software, Funding acquisition, Formal analysis, Data curation, Conceptualization. **Jian Xiong:** Visualization, Validation, Software, Investigation, Formal analysis. **Bibo Zhao:** Validation, Project administration, Methodology, Investigation, Formal analysis, Data curation. **Chuanfu Li:** Visualization, Validation, Supervision, Methodology, Investigation, Funding acquisition. **Senyou Tan:** Supervision, Software, Resources, Methodology, Investigation. **Qiang Kang:** Software, Methodology, Investigation, Funding acquisition. **Hao Xiao:** Visualization, Supervision, Software, Methodology, Investigation, Funding acquisition. **Xiaofeng Tan:** Writing – review & editing, Supervision, Software, Methodology, Investigation, Funding acquisition, Formal analysis, Data curation, Conceptualization. **Gui-long Wu:** Writing – review & editing, Writing – original draft, Visualization, Validation, Supervision, Software, Resources, Project administration, Methodology, Investigation, Funding acquisition, Formal analysis. **Qinglai Yang:** Writing – review & editing, Writing – original draft, Validation, Software, Resources, Investigation, Funding acquisition, Formal analysis, Data curation. **Guodong Chen:** Writing – review & editing, Writing – original draft, Validation, Software, Project administration, Methodology, Investigation, Funding acquisition, Formal analysis, Data curation, Conceptualization.

## Declaration of competing interest

We declare that we do not have any commercial or associative interest that represents a conflict of interest in connection with the work submitted.

## Data Availability

Data will be made available on request.
