# Supplementary material for: Conversion therapy strategy: A novel GPC3-targeted multimodal organic phototheranostics platform for mid-late-stage hepatocellular carcinoma
Source: Mater Today Bio. 2025 Jan 2;30:101442. doi: 10.1016/j.mtbio.2024.101442 (PMC11762635; doi:10.1016/j.mtbio.2024.101442)
Supplement: Multimedia component 1 [file mmc1.docx]

**Supporting Information**

**Conversion Therapy Strategy: A Novel GPC3-Targeted Multimodal Organic Phototheranostics Platform for** **Mid-late-stage Hepatocellular Carcinoma**

Fan Wu[^a^](#单位a)^,^[^b^](#单位b)^,^[^c^](#单位c), Xin Kuang[^a^](#单位a)^,^[^b^](#单位b)^,^[^c^](#单位c), Sanlin Deng[^a^](#单位a)^,^[^b^](#单位b)^,^[^c^](#单位c), Shuo Qi[^a^](#单位a)^,^[^b^](#单位b), Jian Xiong[^a^](#单位a)^,^[^b^](#单位b)^,^[^c^](#单位c), Bibo Zhao[^a^](#单位a)^,^[^b^](#单位b)^,^[^c^](#单位c), Chuanfu Li[^a^](#单位a)^,^[^b^](#单位b)^,^[^c^](#单位c), Senyou Tan[^a^](#单位a)^,^[^b^](#单位b), Qiang Kang[^b^](#单位a), Hao Xiao[^b^](#单位a), Xiaofeng Tan[^b^](#单位a)[^****^](#通讯4), Gui-long Wu[^a^](#单位a)^,^[^b^](#单位b)^,^[^c^](#单位c)[^***^](#通讯3), Qinglai Yang [^a^](#单位a)^,^[^b^](#单位b)^,^[^c^](#单位c)[^**^](#通讯2), Guodong Chen [^a^](#单位a)^,^[^b^](#单位b)^,^[^c^](#单位c)^,d^[^*^](#通讯1)

^a^*Department of Hepatopancreatobiliary Surgery, The First Affiliated Hospital, Hengyang Medical School, University of South China, Hengyang, Hunan, 421001, China.*

^b^*Center for Molecular Imaging Probe Hengyang Medical School, University of South China, Hengyang, Hunan, 421001, China.*

^c^*Hunan Engineering Research Center for Early Hengyang Medical School, University of South China, Hengyang, Hunan, 421001, China.*

*^d^Department of general Surgery, Turpan City People's Hospital, Tulufan 838000, China.*

*^*^*Corresponding authors. Department of Hepatopancreatobiliary Surgery, The First Affiliated Hospital, Hengyang Medical School, University of South China, 28 West Changsheng Road, Hengyang, Hunan, 421001, China.

*^**^*Corresponding authors.

*^***^*Corresponding authors.

*^****^*Corresponding authors.

*E-mail addresse*s: [chenguodong@usc.edu.cn](mailto:chenguodong@usc.edu.cn) (G. Chen), [qingyu513@usc.edu.cn](mailto:qingyu513@usc.edu.cn) (Q. Yang), [2018000014@usc.edu.cn](mailto:2018000014@usc.edu.cn) (G. Wu), [tanxiaofeng@usc.edu.cn](mailto:tanxiaofeng@usc.edu.cn) (X. Tan).

Table

[**Scheme S1**. Synthetic route of IGD NPs 6](#_Toc177582023)

[**Fig. S1.** ^1^H NMR of compound 1. 14](#_Toc177582024)

[**Fig. S2.** ^13^C NMR of compound 1. 14](#_Toc177582025)

[**Fig. S3.** HRMS NMR of compound 1 15](#_Toc177582026)

[**Fig. S4.** ^1^H NMR of compound 2. 16](#_Toc177582027)

[**Fig. S5.** ^13^C NMR of compound 2. 16](#_Toc177582028)

[**Fig. S6.** HRMS of compound 2. 17](#_Toc177582029)

[**Fig. S7.** ^1^H NMR of compound 3. 17](#_Toc177582030)

[**Fig. S8.** ^13^C NMR of compound 3. 18](#_Toc177582031)

[**Fig. S9.** ^1^H NMR of IGD. 18](#_Toc177582032)

[**Fig. S10.** ^13^C NMR of IGD. 19](#_Toc177582033)

[**Fig. S11.** The SEC traces results show that different molecules. 19](#_Toc177582034)

[**Fig. S12.** Zeta potential of IGD NPs, Error bars: mean ± SD (n = 3). 20](#_Toc177582035)

[**Fig. S13.** (A) Photothermal warming curves of IR820 and IGD NPs. (B) Photothermal warming curves of IGD NPs with different power. 21](#_Toc177582036)

[**Fig. S14.** After 5 min of laser irradiation, the DCF fluorescence values in aqueous solution of PBS, IR820, and IGD NPs (488 nm). 22](#_Toc177582037)

[**Fig. S15.** (A, B) In vivo FLI and fluorescence values of subcutaneous tumor-bearing mice at different time points after tail vein injection of free ICG and IGD NPs probes. Error bars: mean ± SD (n = 4). 23](#_Toc177582038)

[**Fig. S16.** Signal values of in vivo magnetic resonance T1-weighted imaging in mice at different time points.ST: subcutaneous tumors, PIS: in situ tumors, Error bars: mean ± SD (n =4), ***p < 0.001. 24](#_Toc177582039)

[**Fig. S17.** Surgical excision procedure. 25](#_Toc177582040)

[**Fig. S18.** Pre- and post-treatment bioluminescence imaging for monitoring hepatocellular carcinoma in situ in different groups. 26](#_Toc177582041)

[**Fig. S19.** Difference plots of routine hematological and biochemical assays in mice of different treatment groups, Error bars: mean ± SD (n = 4). 27](#_Toc177582042)

[**Fig. S20.** H&E staining of each organ in mice of different treatment groups; scale bar: 200 μm. 28](#_Toc177582043)

[**Table S1.** Molecule weight determined by SEC-MALLS. 29](#_Toc177582044)

*General measurements*

Nuclear magnetic resonance (NMR) spectra were recorded on an AVANCE NEO 500 spectrometer (Bruker, Germany) using CDCl_3_ as the internal reference. High-resolution mass spectra (HRMS) and size exclusion chromatography (SEC) were obtained on a Thermo Scientific Q Exactive Combined quadrupole Orbitrap mass spectrometer (Thermo Fisher Scientific Co, USA) in a Positive ion mode. Confocal laser scanning microscopy (CLSM) images were collected on Zeiss LSM880 (Zeiss, Germany). Transmission electron microscopy (TEM) images were performed on an HT7800 transmission electron microscope (Hitachi Electronics, accelerating voltage 80 kV). The size distributions of the NPs were obtained from Nano-ZS90 (Malvern, China). The size distribution of IR820-GPC3-Gd NPs (IGD NPs) was measured by a nanometer particle size potentiometer (Malvern Nano-ZS90). Fluorescence spectra were performed on a Thermo Scientific Lumina fluorescence spectrometer (Thermo Fisher Scientific Co, USA), Ultra violet-visible-near infrared (UV-Vis-NIR) absorption spectra were measured by a UA-3200S spectrometer (MAPADA, China). The pathological sections were observed via Pannoramic DESK (3D HISTECH, HUN). Fluorescence spectroscopy was measured with the In Vivo Imaging System for Small Animals (IVIS Spectrum, USA). MRI signal using 3.0T ultra-high field magnetic resonance (SIEMENS, Germany). Temperature evolution curves were tested by an infrared thermal imaging camera (Fotric 225s, China) upon irradiation with an 808 nm near-infrared (NIR) laser (1.0 W/cm^2^, MDL-XF-808nm/10W, Changchun New Industries Optoelectronics Technology Co, Ltd.).

Synthesis and characterization of IGD NPs

**Scheme S1**. Synthetic route of IGD NPs

**Compound 1**: 2.0 g (5.0 mmol) of phloroglucinol and 6.4 g (31.7 mmol) of 1,3-Dibromopropane were added to a 100 mL dry round-bottomed flask, and 4.4 g (31.8 mmol) of K_2_CO_3_ and 10 mL of N,N-Dimethylformamide (DMF) were added sequentially, and the reaction was carried out for 12 h at room temperature. The product was extracted with equal volumes of water and ethyl acetate (EA) to remove excess K_2_CO_3_ and DMF. Rotary evaporation removed the EA, and then the product was separated by adding it to a chromatographic column filled with silica gel powder. After separation using a solution of petroleum ether (PE)/EA = 8, rotary evaporation yielded 3.8 g (10.3 mmol) (65.0%) of white solid compound 1. ^1^H NMR (500 MHz, CDCl_3_) δ 6.01 (t, *J* = 2.1 Hz, 1H), 5.96 (d, *J* = 2.1 Hz, 2H), 3.98 (dd, *J* = 7.1, 4.5 Hz, 6H), 3.52 (t, *J* = 6.4 Hz, 6H). ^13^C NMR (126 MHz, CDCl_3_) δ 160.75, 157.38, 95.23, 95.05, 94.50, 94.30, 77.27, 77.01, 76.76, 65.44, 32.29, 29.90. HRMS (ESI) calcd for C_12_H_17_O_3_Br_2_^+^, ([M+H^+^]) 368.9512, Found 368.9518.

**Compound 2**: The crude product of the reaction was obtained by adding 0.8 g (2.2 mmol) Compound 1, 0.4 g (6.2 mmol) NaN₃, and 10 mL of DMF to a 100 mL dry round bottom flask and stirring overnight at 70℃ in the absence of water and oxygen. The reaction was separated using a solution chromatography column with PE/EA = 4, and 0.6 (2.1 mmol) (94.5%) of clear light yellow liquid product was obtained after rotary evaporation and drying. Then 0.2 g of IR820, 35 mg of NaH, and 10 mL of DMF were added and the reaction was carried out at low temperature for 3 h under anhydrous and oxygen-free conditions. The product was filtered through a hydrophobic filter centrifuged twice with an equal ratio of PE/EA = 1, and dried to give a final 0.2 g (0.2 mmol) (77.0%) of dark green solid compound 2. ^1^H NMR (500 MHz, CD_3_OD_SPE) δ 8.09 (s, 3H), 7.94 (d, *J* = 2.8 Hz, 4H), 7.57 (d, *J* = 3.7 Hz, 4H), 7.43 (s, 2H), 7.33 (s, 1H), 7.14 (s, 1H), 6.21 (d, *J* = 14.3 Hz, 4H), 4.25 (d, *J* = 4.5 Hz, 2H), 3.45 (d, *J* = 5.4 Hz, 2H), 3.31 (dd, *J* = 10.5, 8.9 Hz, 16H), 2.89 (s, 4H), 2.79 (d, *J* = 5.8 Hz, 4H), 2.03 – 1.94 (m, 18H). ^13^C NMR (500 MHz, CD_3_OD_SPE) δ 160.75, 157.38, 95.23, 95.05, 94.50, 94.30, 77.27, 77.01, 76.76, 65.44, 32.29, 29.90. HRMS (ESI) calcd for C_58_H_65_O_9_N_8_S_2_^+^, ([M+H^+^]) 1081.4321, Found 1081.4373.

**Compound 3**: 20 mg (18.1 μmol) of compound 2 with 65 mg (18.3 μmol) of GPC3 peptide was added to a 25 mL dry flask. 0.46 mg (2.3 μmol) of sodium ascorbate and 0.24 mg (1.3 μmol) of copper acetate were added to a centrifuge tube of 1 mL of water and 1 mL of isopropanol, respectively, and the centrifuge tube was shaken using an ultrasonic cleaner for 5 min before being added to the reaction flask and stirred for 3 h at anhydrous and anaerobic and 70℃. The reaction product was then dried and lyophilized. The reaction product was dialyzed through a 2 kd dialysis bag for 24 h and lyophilized to give a final 65 mg (14.1 μmol) (78.1%) of black solid compound 3. ^1^H NMR (500 MHz, CD_3_OD_SPE) δ 8.26 (d, *J* = 8.5 Hz, 3H), 7.99 (d, *J* = 10.7 Hz, 4H), 7.62 (s, 4H), 7.51 (s, 2H), 7.22 (s, 1H), 7.10 (s, 1H), 6.21 (d, *J* = 14.0 Hz, 4H), 4.22 (d, *J* = 51.9 Hz, 18H), 3.87 (s, 188H), 2.73 (d, *J* = 50.8 Hz, 18H). ^13^C NMR (500 MHz, CD_3_OD_SPE) δ 160.75, 157.38, 95.23, 95.05, 94.50, 94.30, 77.27, 77.01, 76.76, 65.44, 32.29, 29.90.

**IGD**: Using the same reaction conditions and purification method as for compound 3, IR820-GPC3 was reacted with 10 mg (16.3 μmol) of DOTA-Gd, resulting in 68 mg (14.5 μmol) (92.3%) of black solid IGD NPs. ^1^H NMR (500 MHz, CD_3_OD_SPE) δ 8.26 (s, 3H), 7.98 (s, 4H), 7.62 (s, 8H), 6.21 (s, 4H), 4.22 – 3.33 (m, 287H), 2.91 – 2.65 (m, 21H). ^13^C NMR (500 MHz, CD_3_OD_SPE) δ 160.75, 157.38, 95.23, 95.05, 94.50, 94.30, 77.27, 77.01, 76.76, 65.44, 32.29, 29.90.


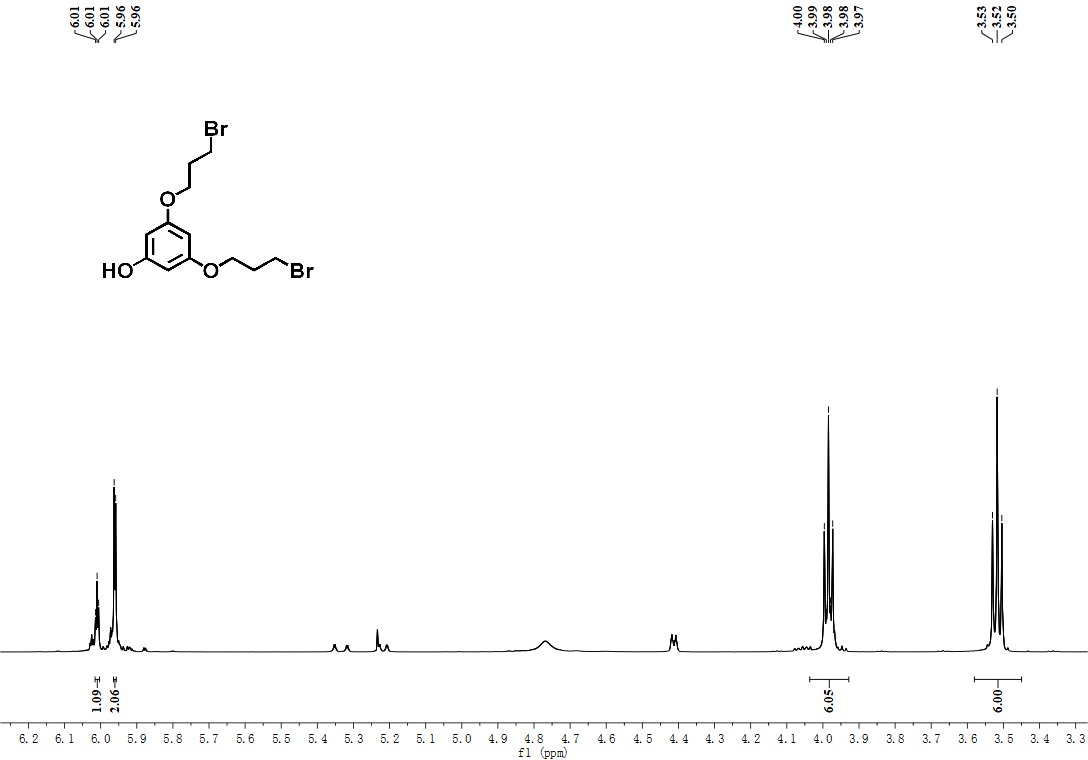


**Fig. S1.** ^1^H NMR of compound 1.


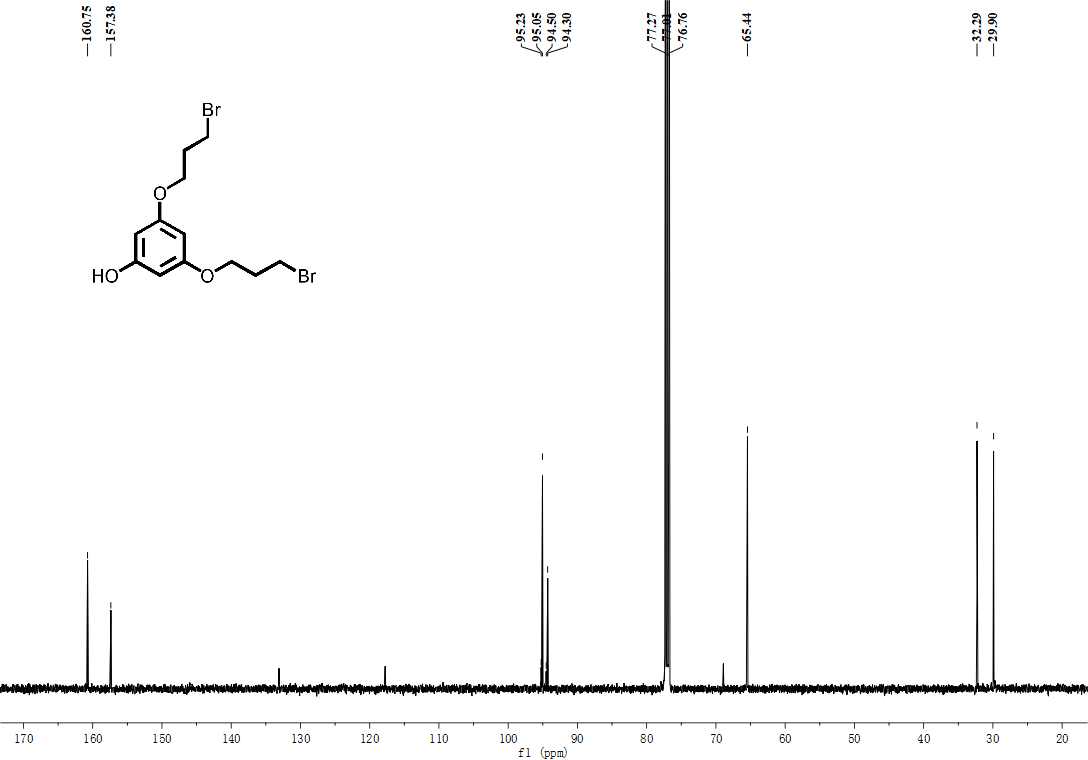


**Fig. S2.** 13C NMR of compound 1.
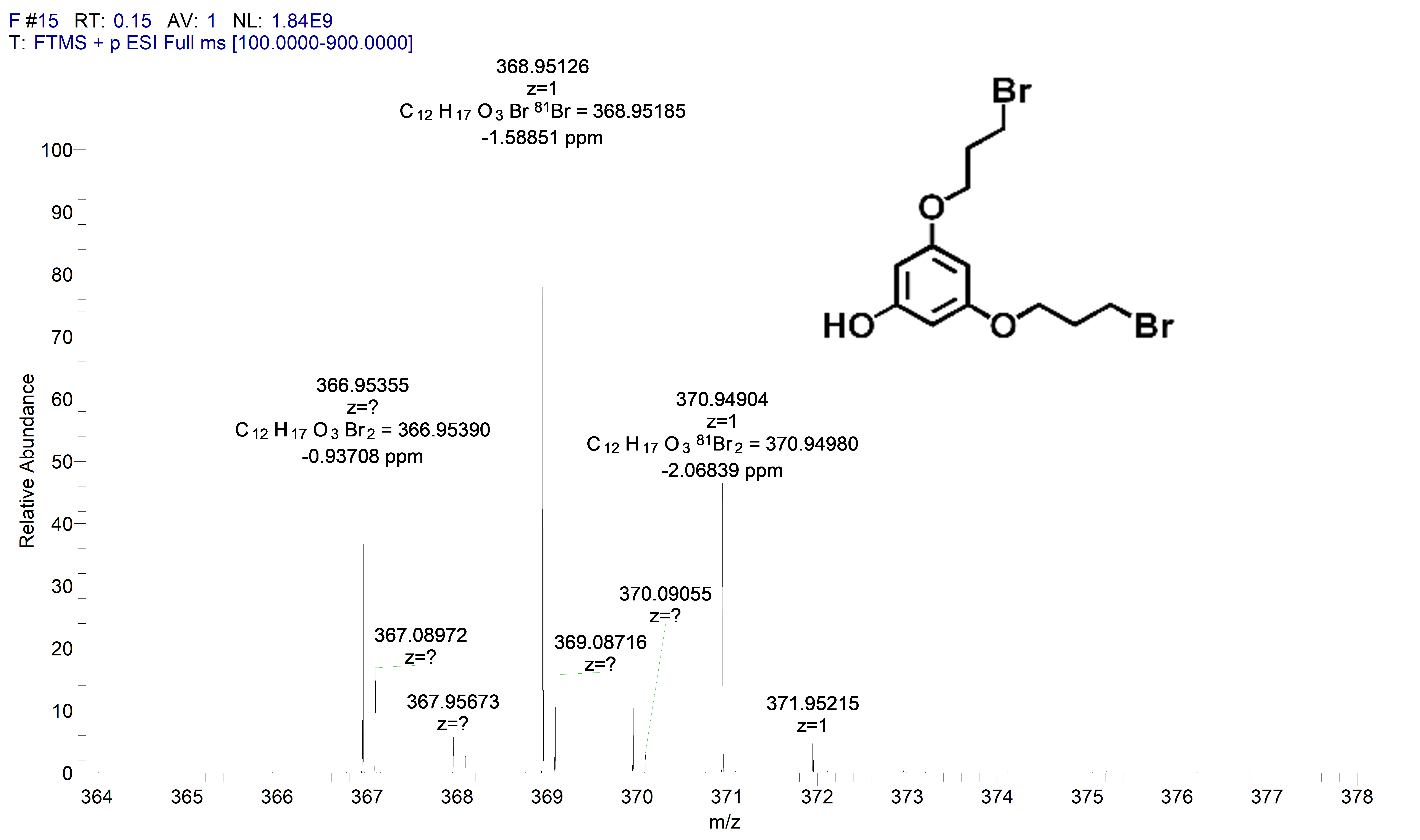


**Fig. S3.** HRMS NMR of compound 1


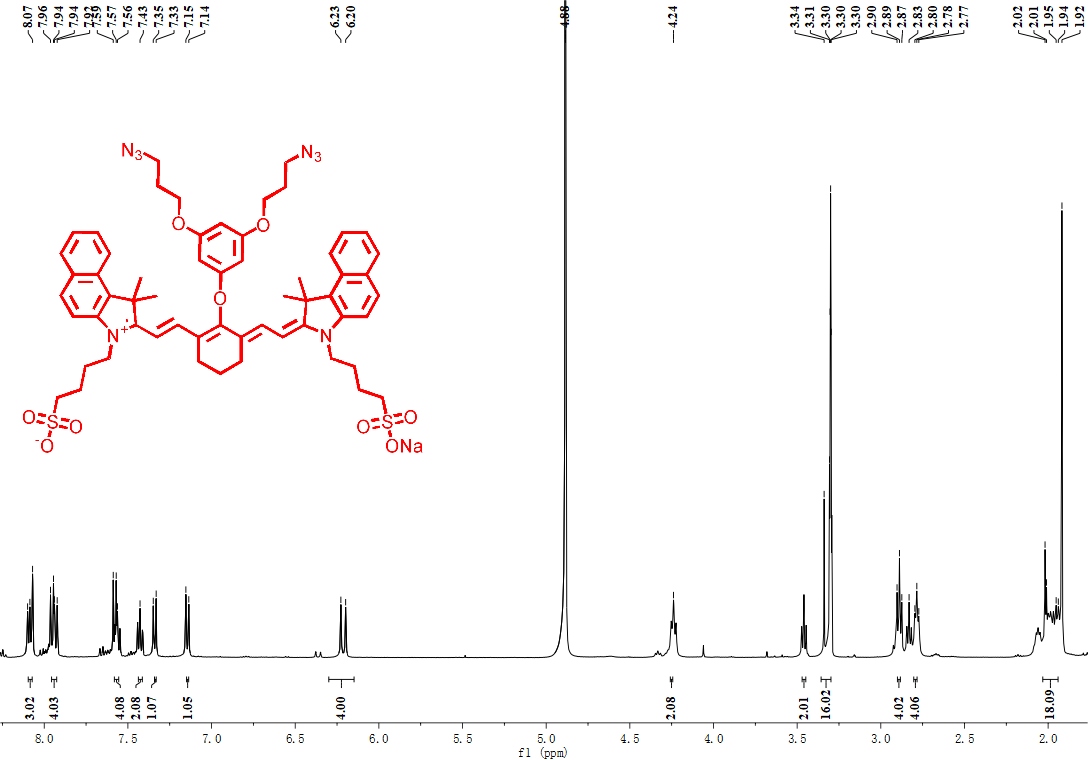


**Fig. S4.** ^1^H NMR of compound 2.


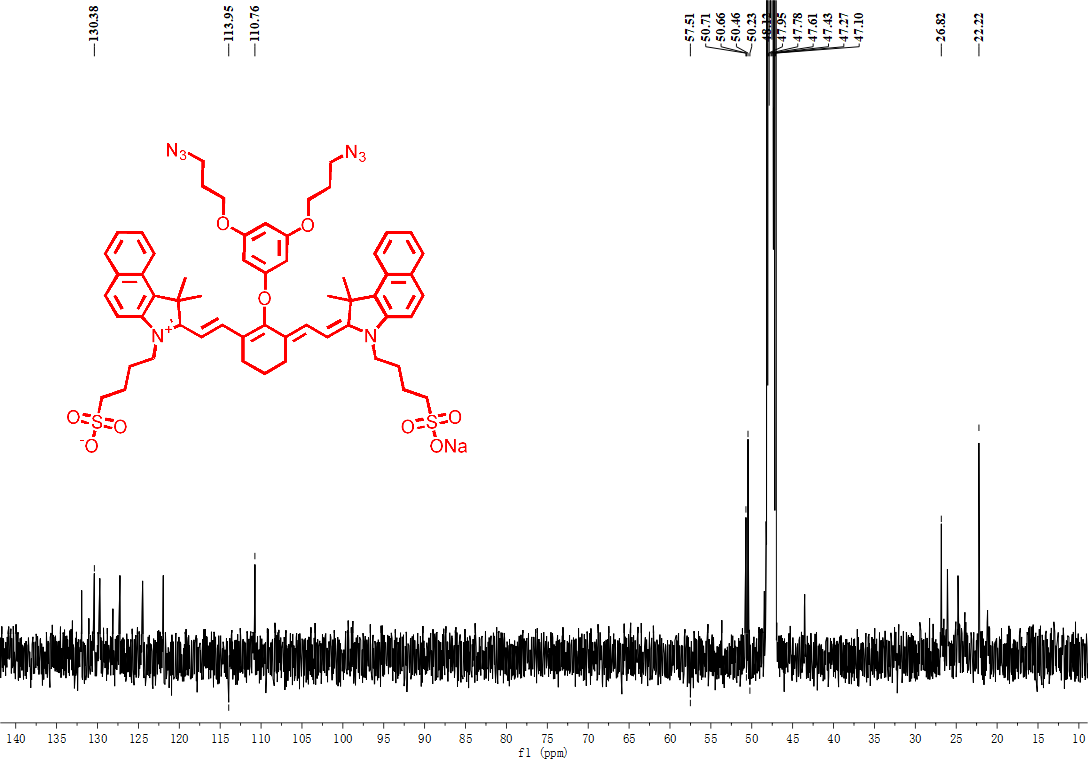


**Fig. S5.** ^13^C NMR of compound 2.


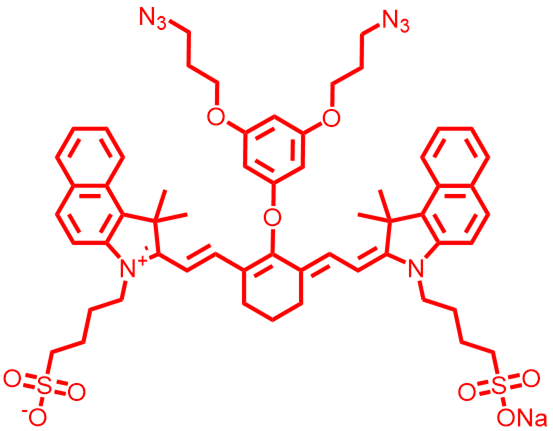


**Fig. S6.** HRMS of compound 2.


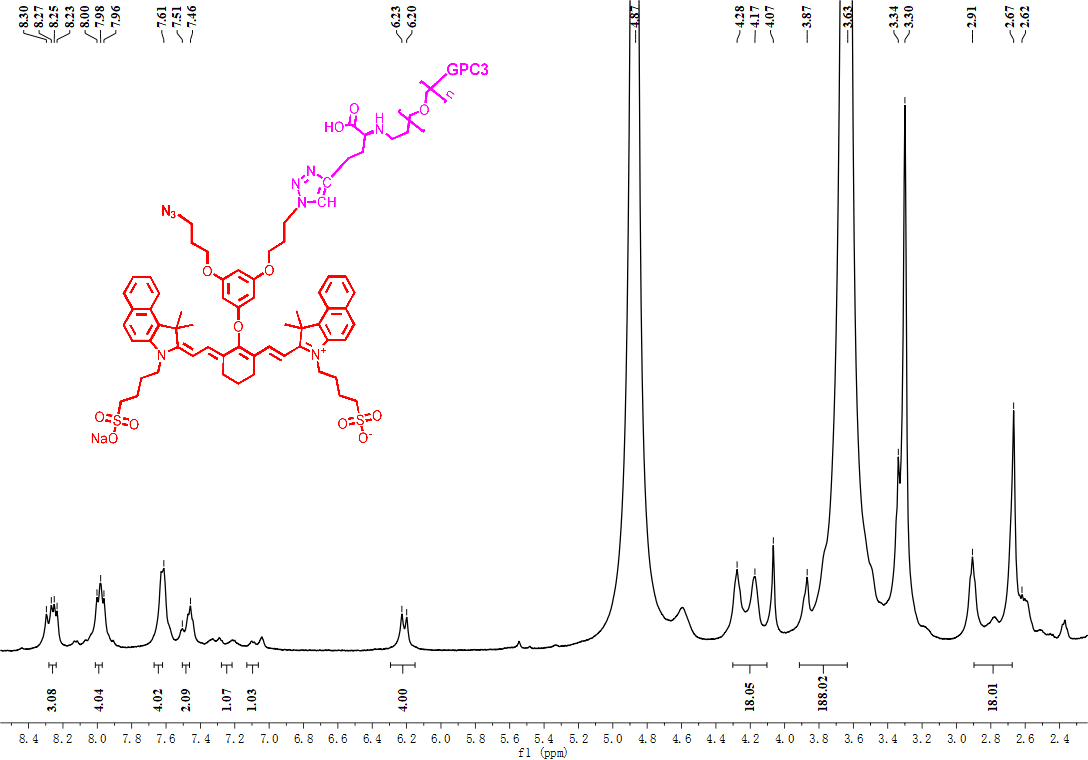


**Fig. S7.** ^1^H NMR of compound 3.


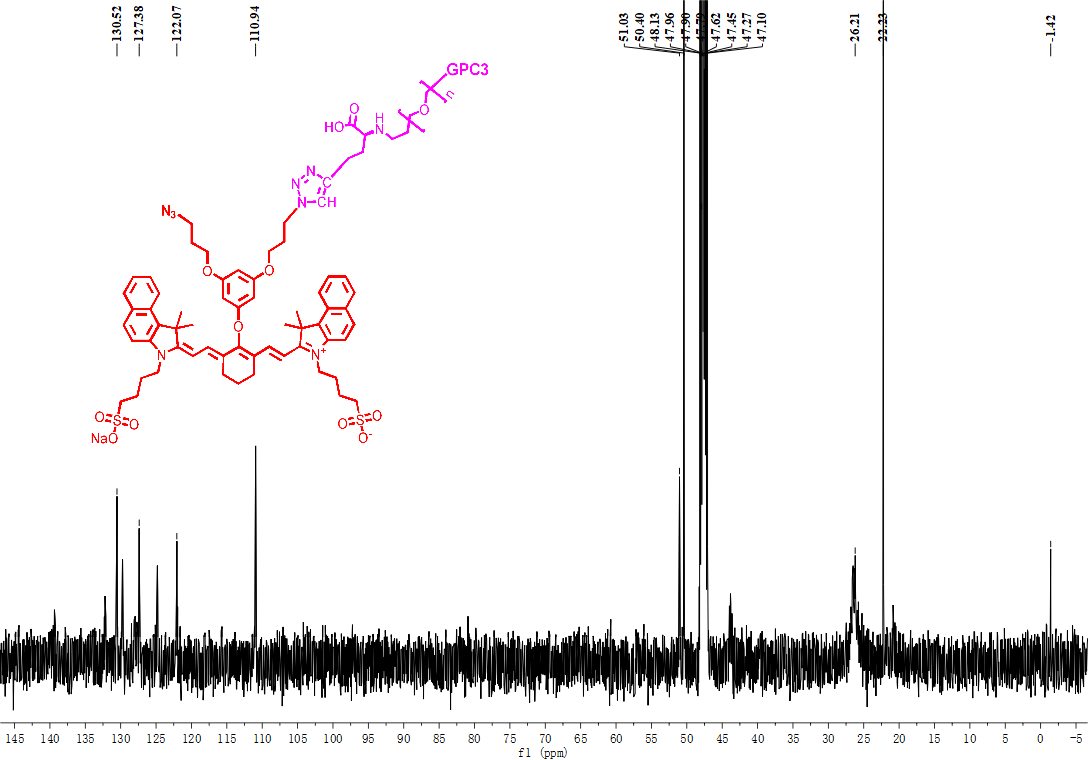


**Fig. S8.** ^13^C NMR of compound 3.


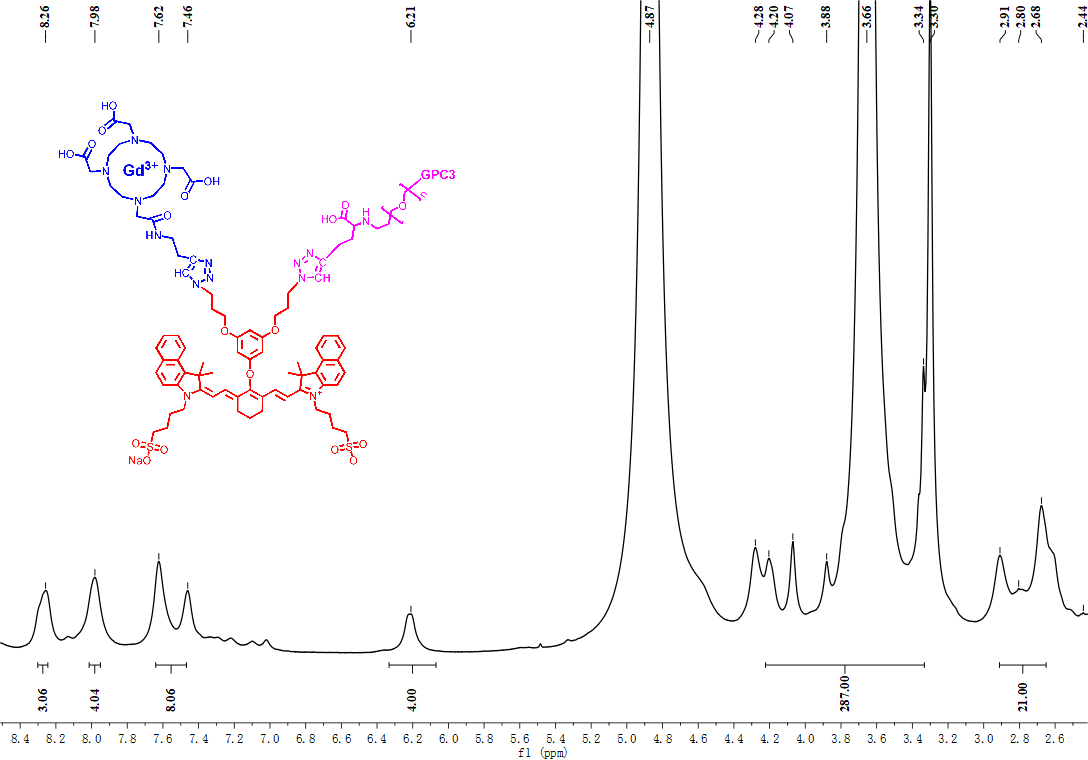


**Fig. S9.** ^1^H NMR of IGD.


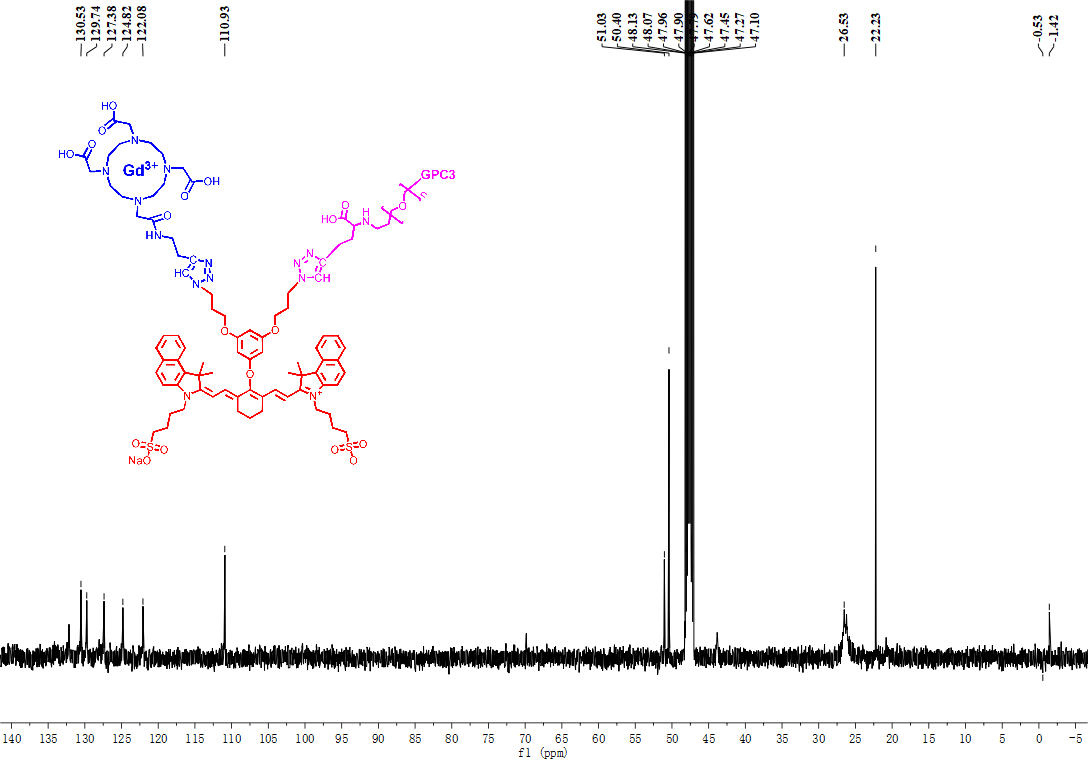


**Fig. S10.** ^13^C NMR of IGD.


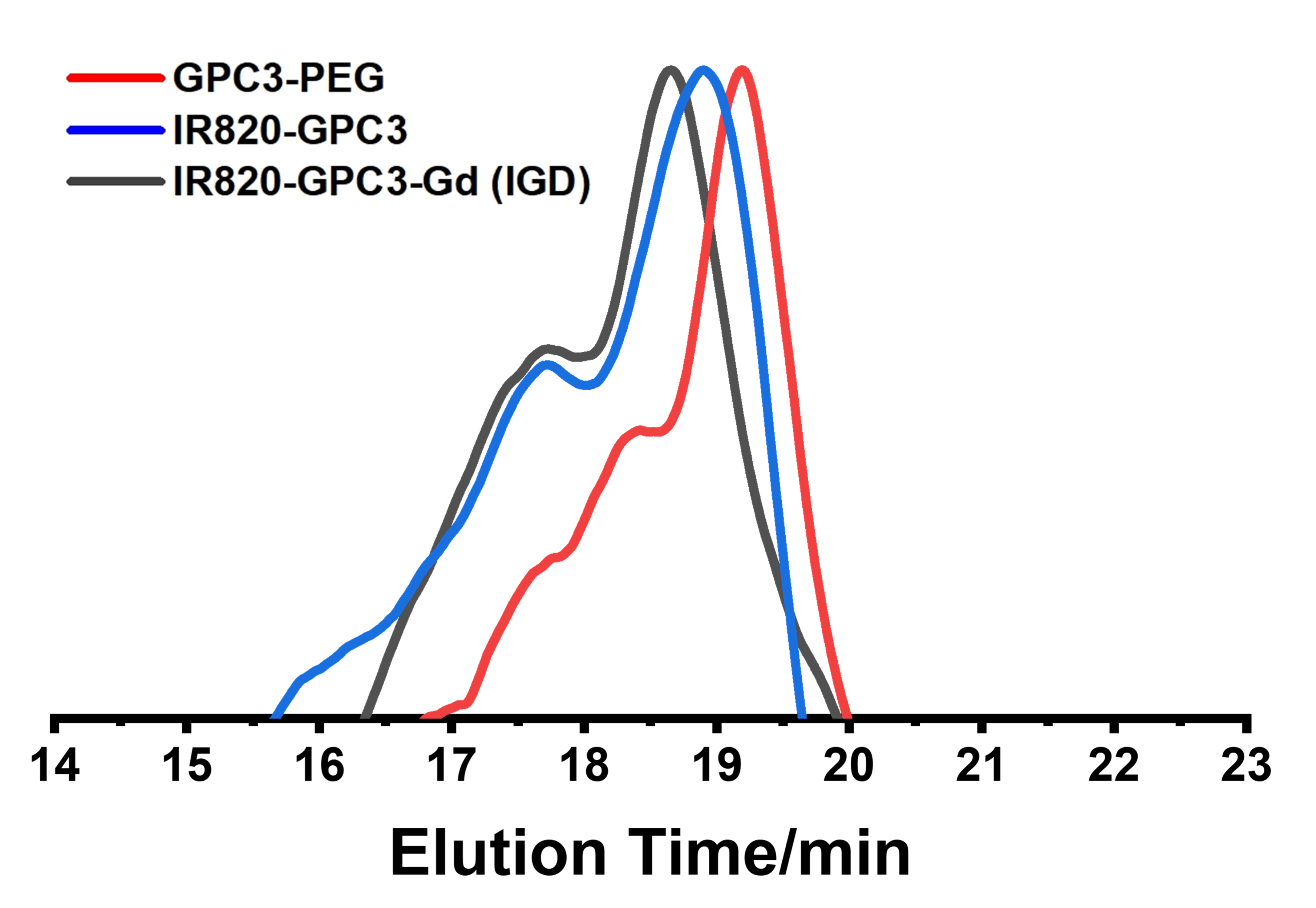


**Fig. S11.** The SEC traces results show that different molecules.


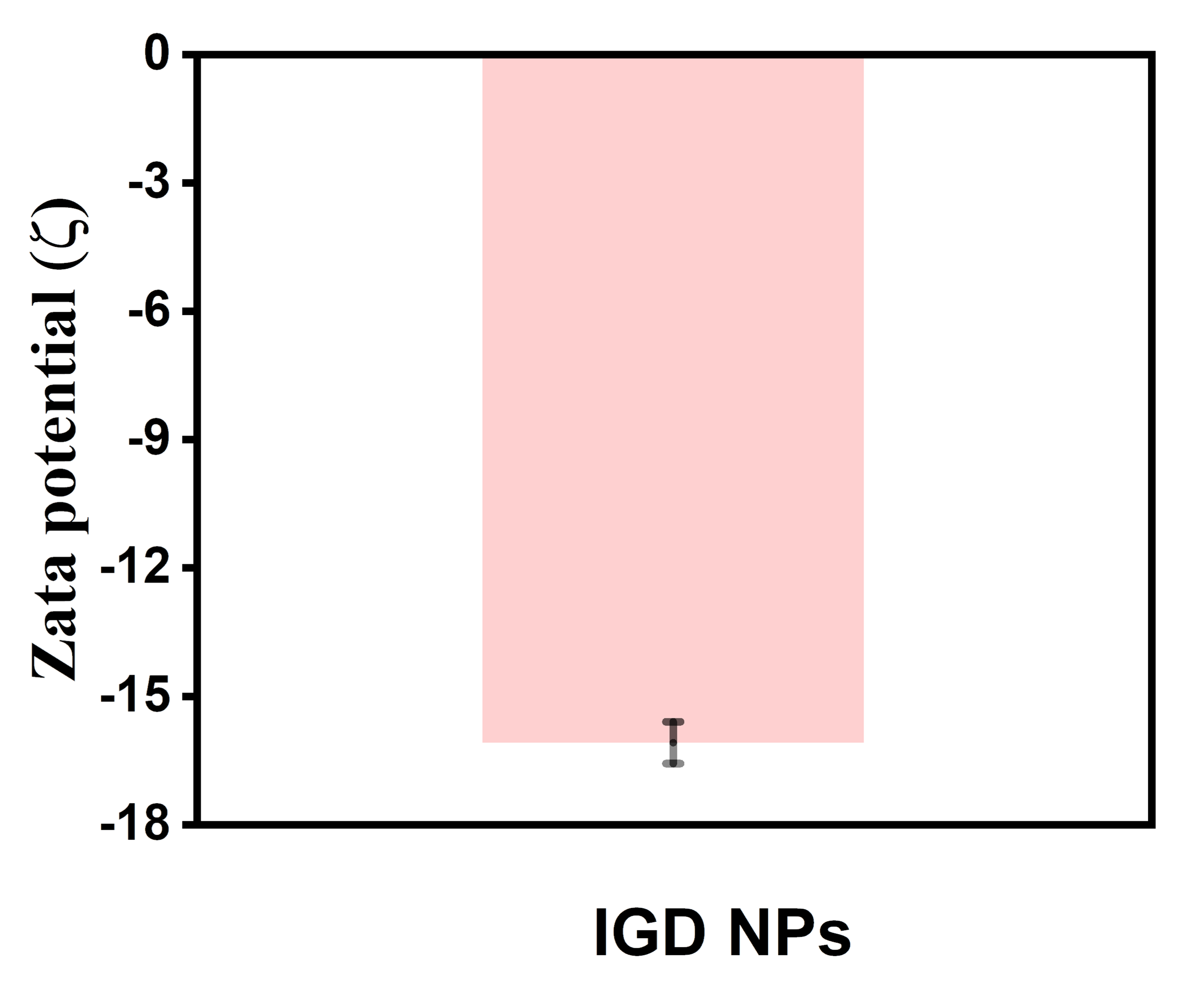


**Fig. S12.** Zeta potential of IGD NPs, Error bars: mean ± SD (n = 3).


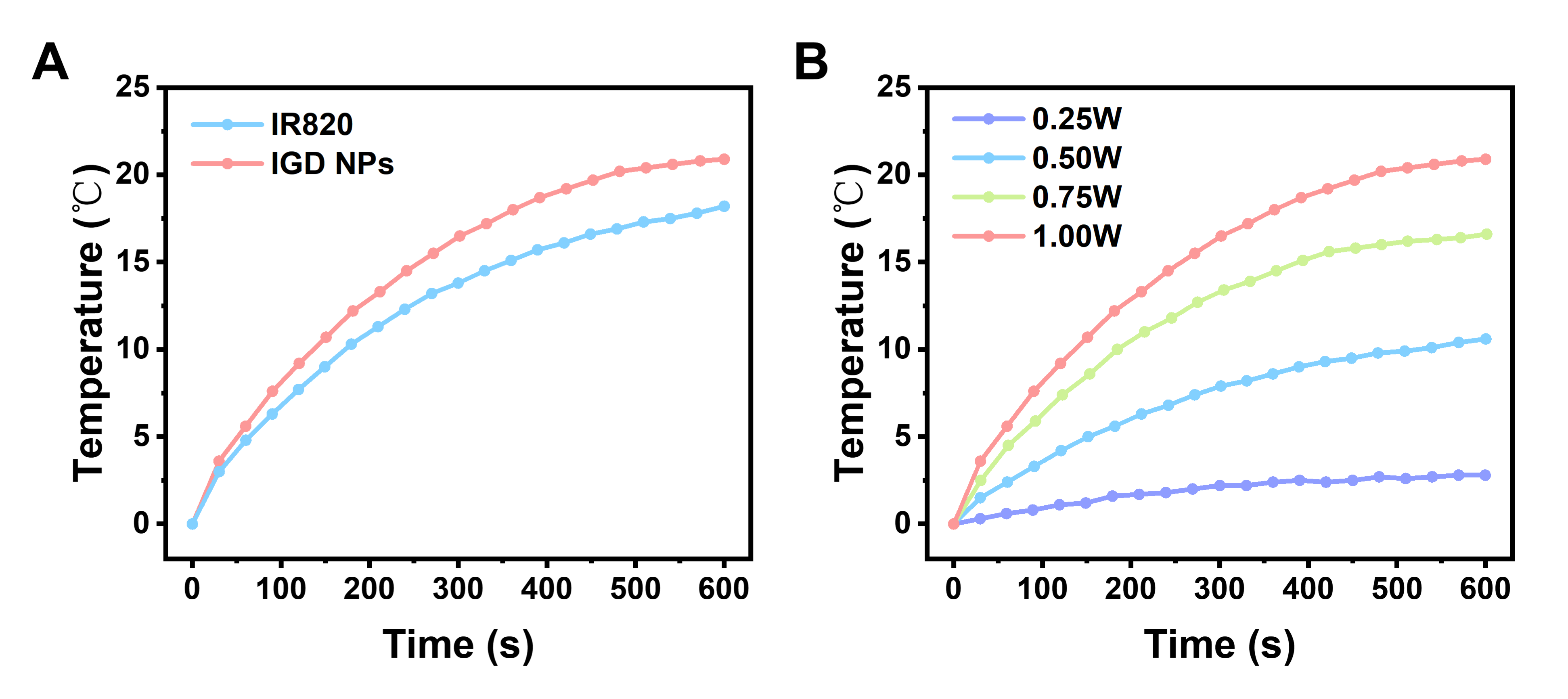


**Fig. S13.** (A) Photothermal warming curves of IR820 and IGD NPs. (B) Photothermal warming curves of IGD NPs with different power.


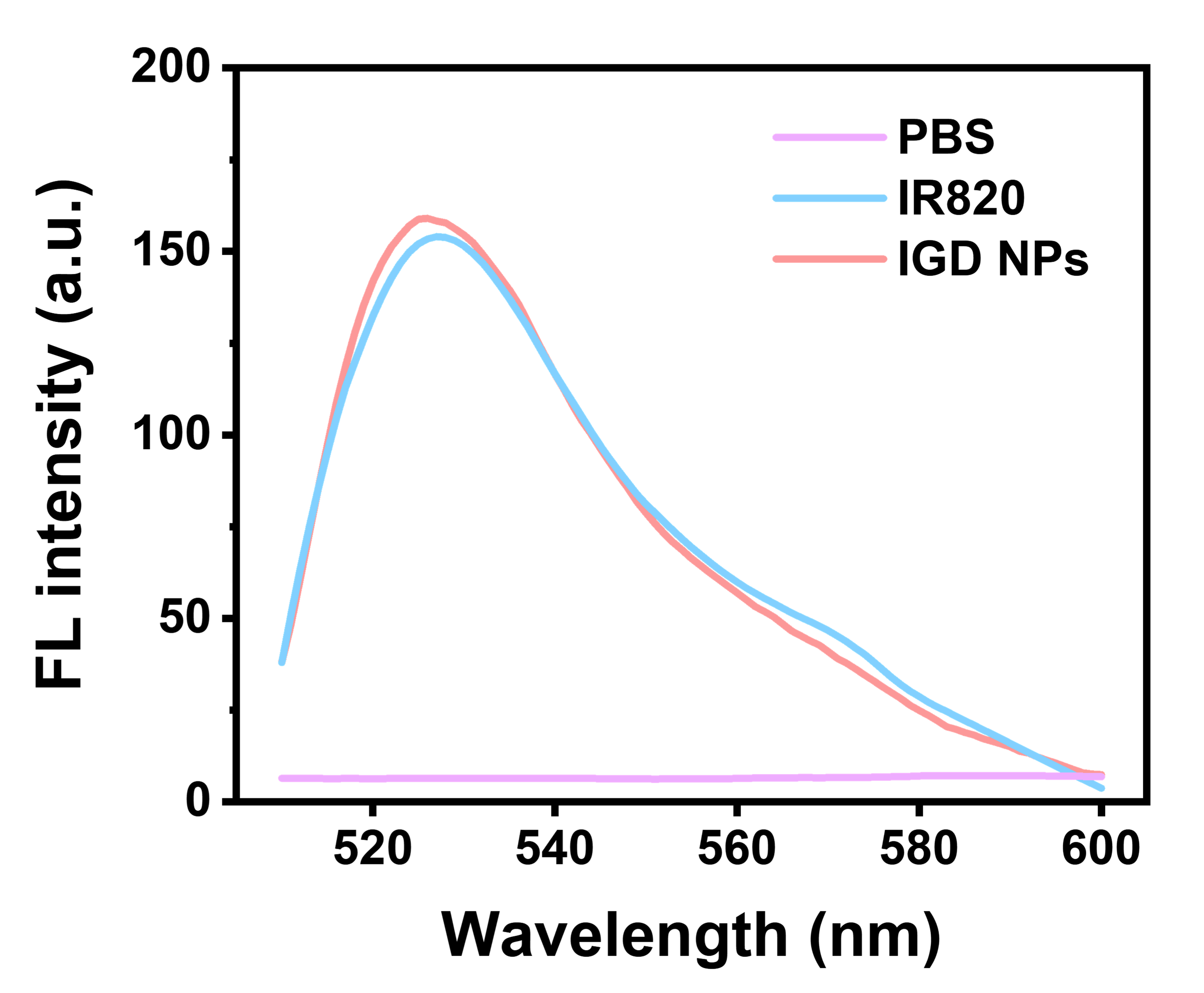


**Fig. S14.** After 5 min of laser irradiation, the DCF fluorescence values in aqueous solution of PBS, IR820, and IGD NPs (488 nm).


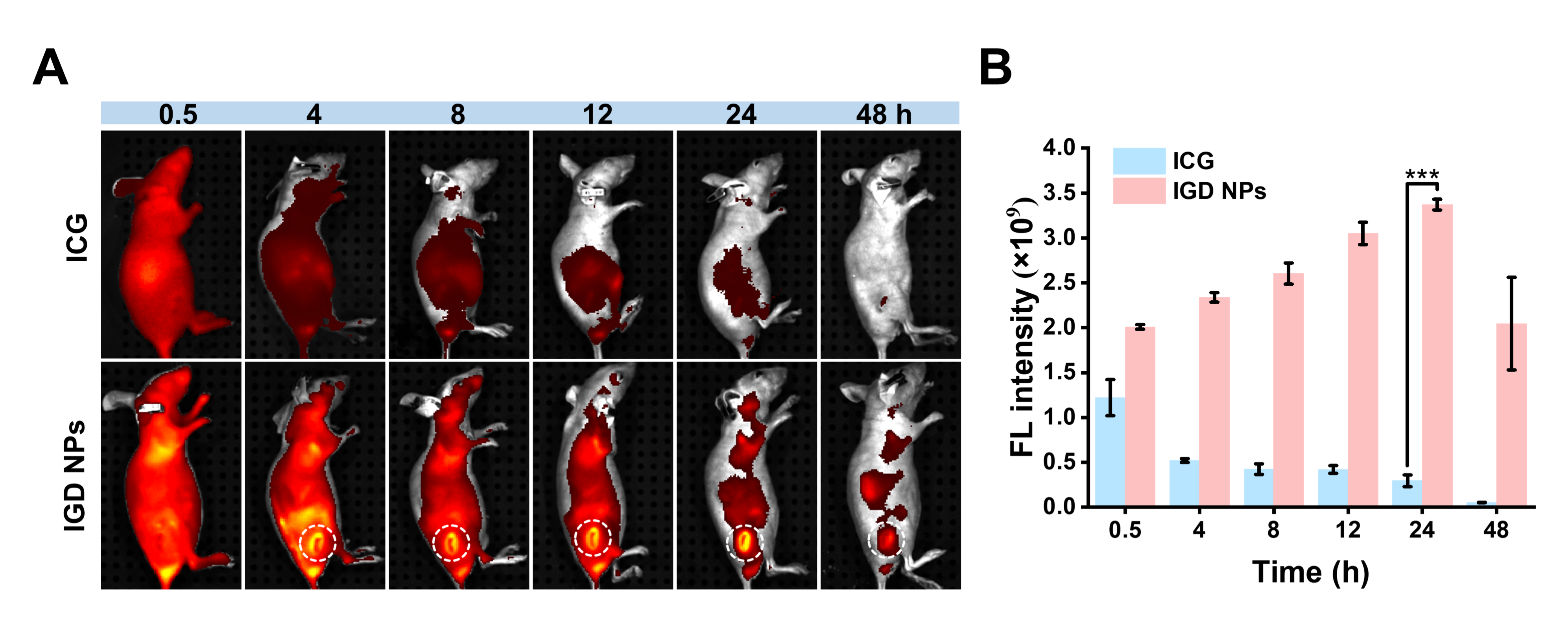


**Fig. S15.** (A, B) In vivo FLI and fluorescence values of subcutaneous tumor-bearing mice at different time points after tail vein injection of free ICG and IGD NPs probes. Error bars: mean ± SD (n = 4).


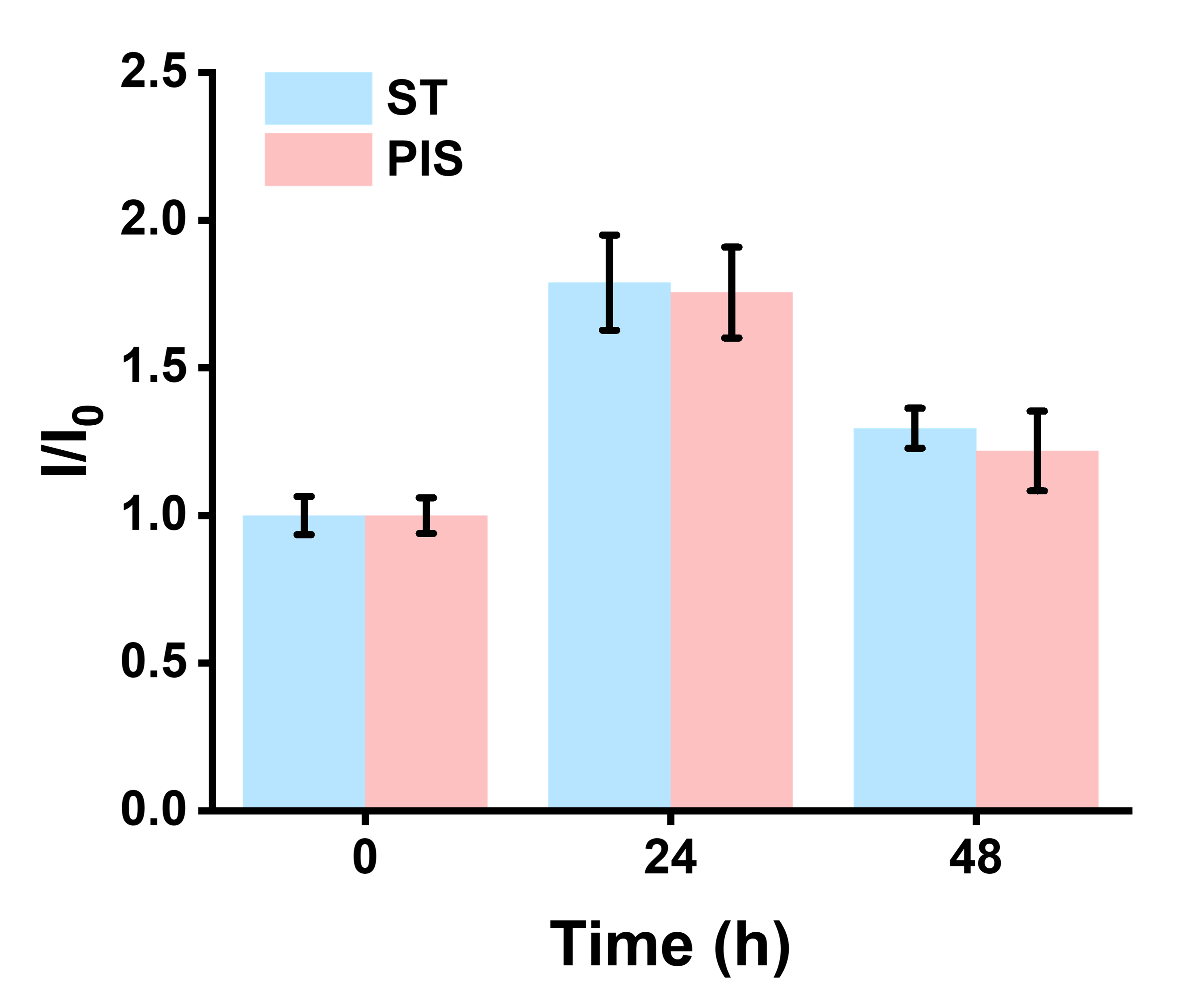


**Fig. S16.** Signal values of in vivo magnetic resonance T1-weighted imaging in mice at different time points.ST: subcutaneous tumors, PIS: in situ tumors, Error bars: mean ± SD (n =4), ***p < 0.001.


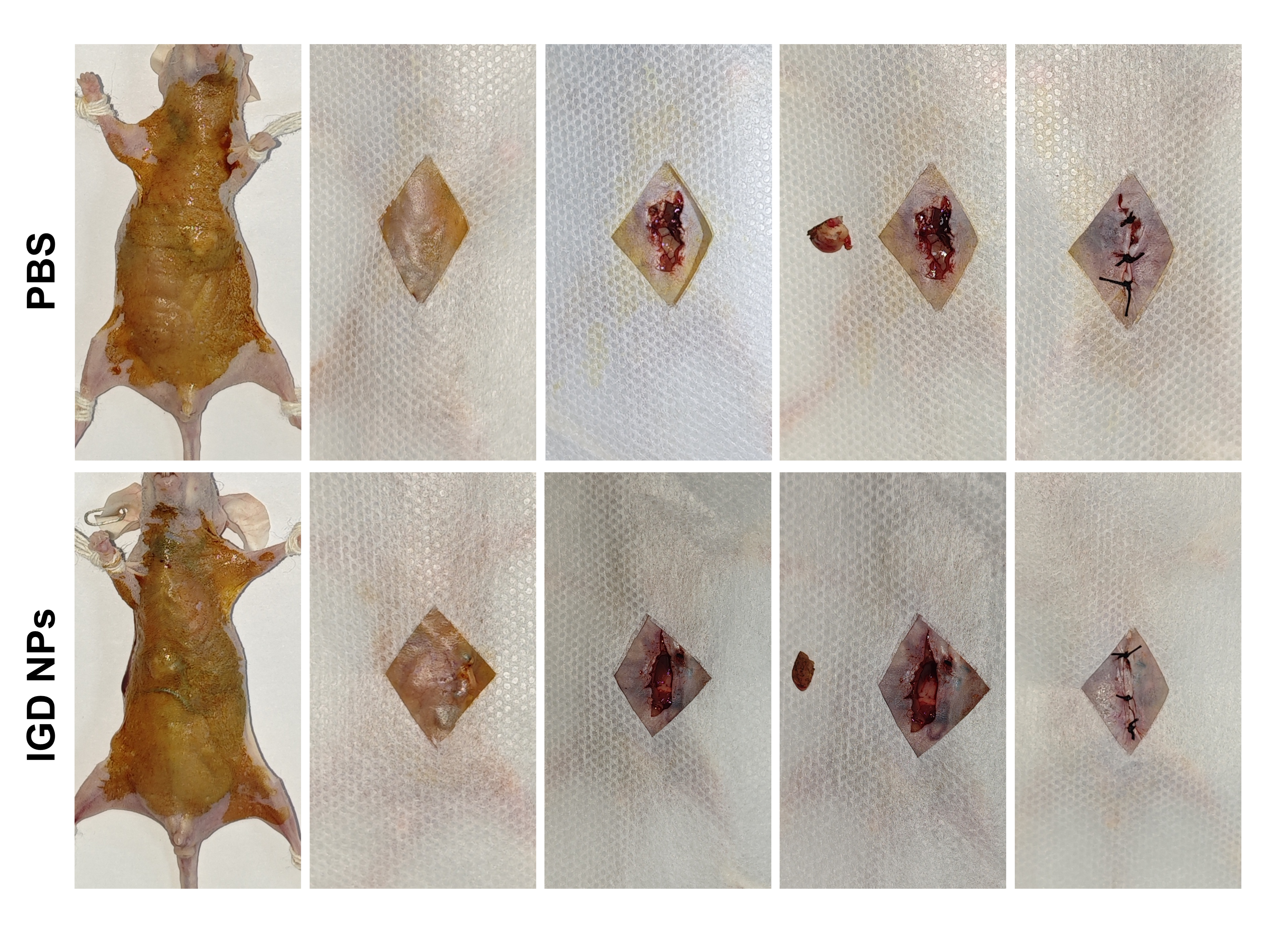


**Fig. S17.** Surgical excision procedure.


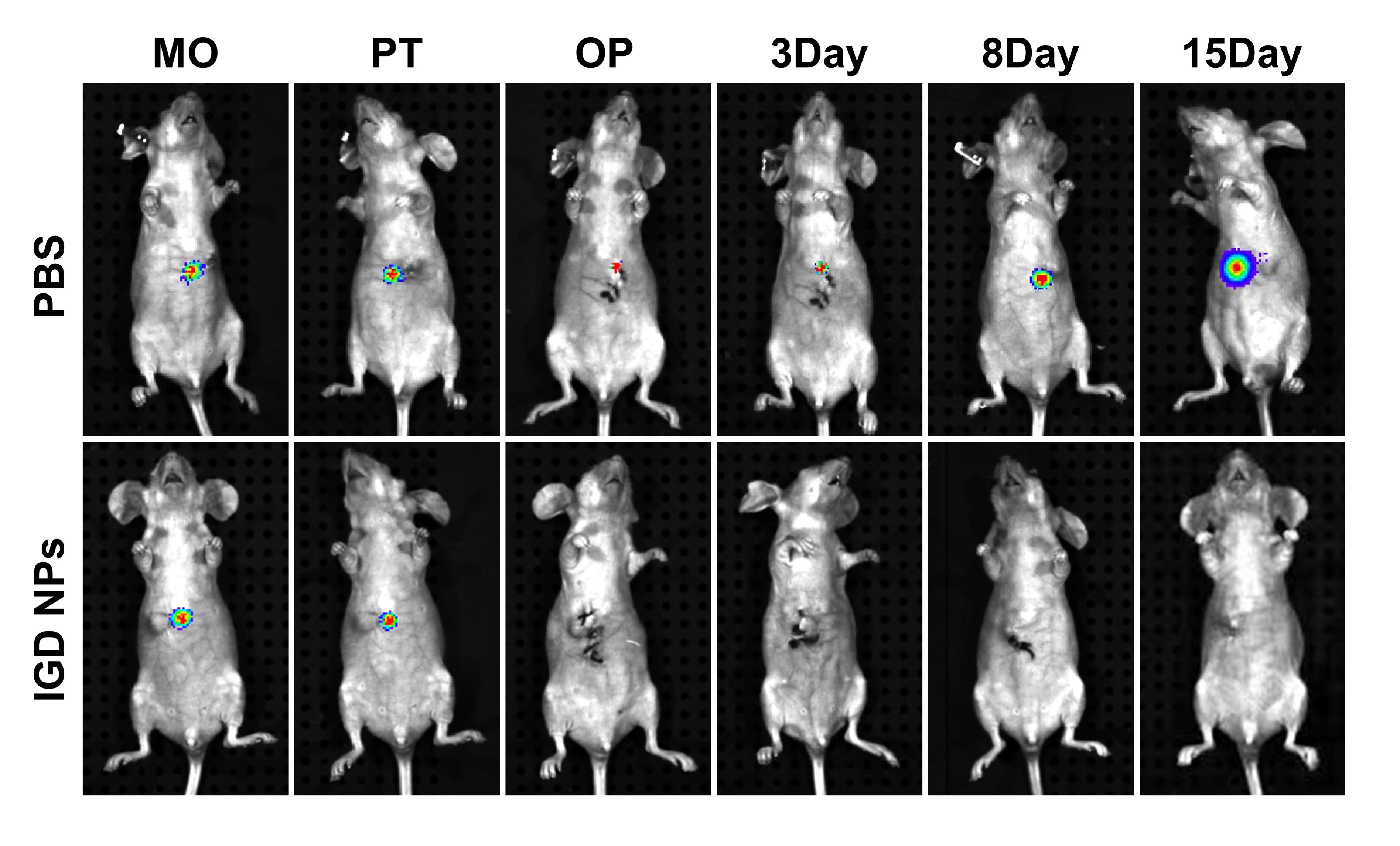


**Fig. S18.** Pre- and post-treatment bioluminescence imaging for monitoring hepatocellular carcinoma in situ in different groups.


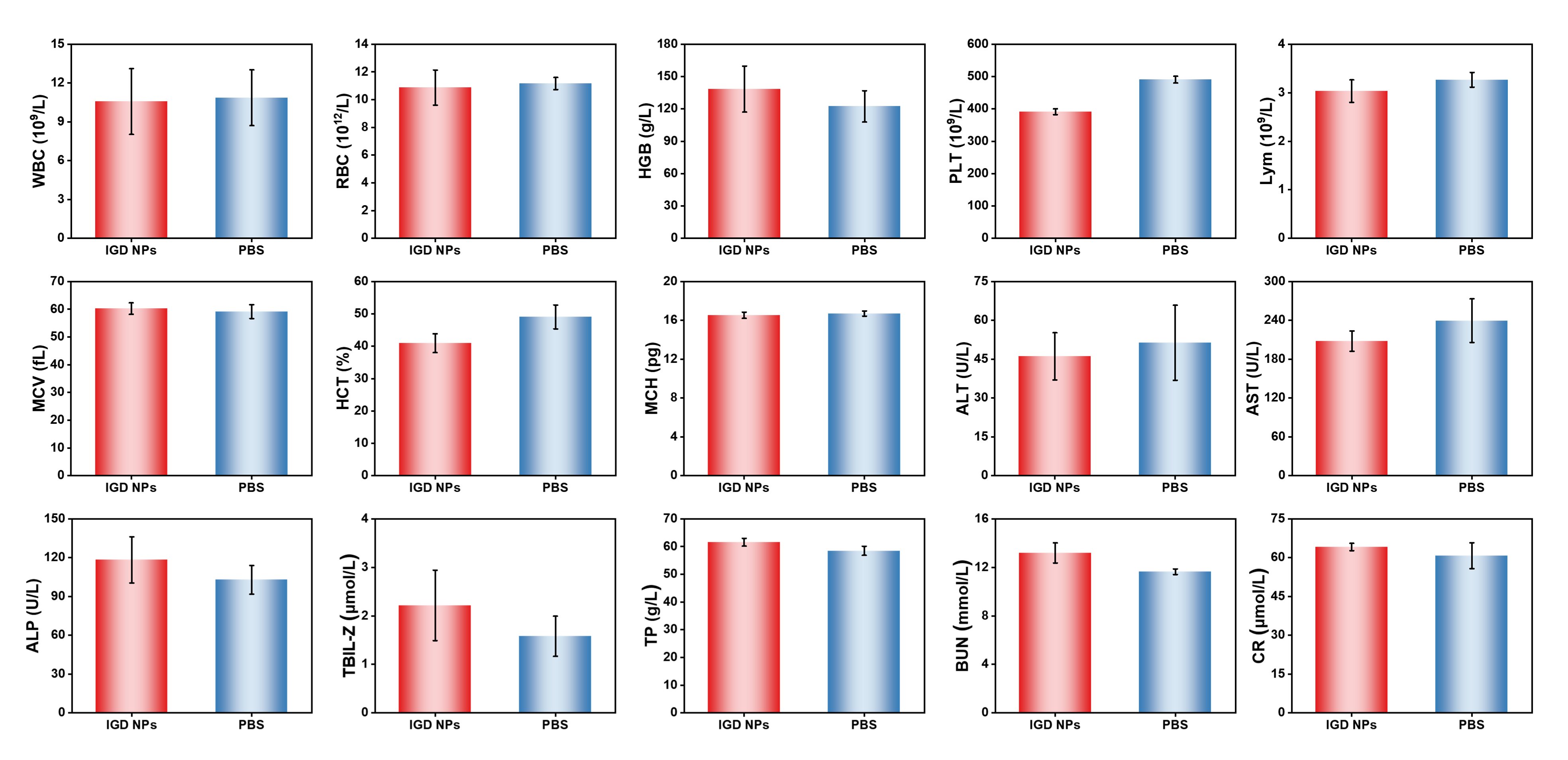


**Fig. S19.** Difference plots of routine hematological and biochemical assays in mice of different treatment groups, Error bars: mean ± SD (n = 4).


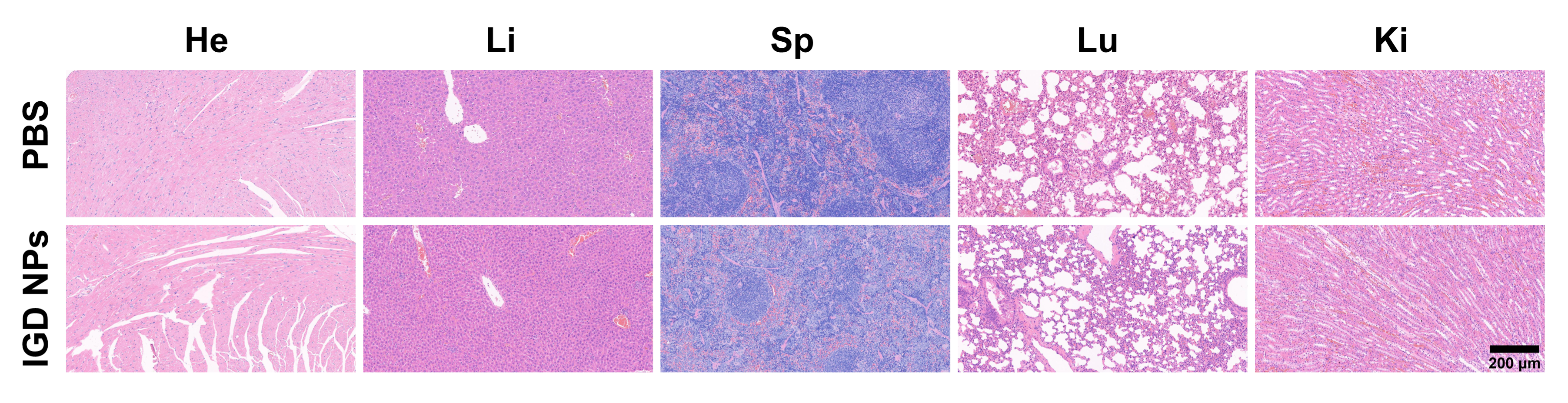


**Fig. S20.** H&E staining of each organ in mice of different treatment groups; scale bar: 200 μm.

**Table S1.** Molecule weight determined by SEC-MALLS.

| Dye | dn/dc (mL/g) | M_n_ (Daltons) | M_w_ (Daltons) | M_w_/M_n_ |
| --- | --- | --- | --- | --- |
| GPC3-PEG2000 | 0.030 | 3200 | 2900 | 1.103 |
| IR820-GPC3 | 0.046 | 4300 | 4100 | 1.048 |
| IR820-GPC3-Gd | 0.042 | 4900 | 4700 | 1.043 |
